# Strength gains after 12 weeks of resistance training correlate with neurochemical markers of brain health in older adults: a randomized control ^1^H-MRS study

**DOI:** 10.1007/s11357-023-00732-6

**Published:** 2023-01-26

**Authors:** Samrat Sheoran, Wouter A. J. Vints, Kristina Valatkevičienė, Simona Kušleikienė, Rymantė Gleiznienė, Vida J. Česnaitienė, Uwe Himmelreich, Oron Levin, Nerijus Masiulis

**Affiliations:** 1 Department of Health Promotion and Rehabilitation, Lithuanian Sports University, 44221 Kaunas, Lithuania; 2 Faculty of Kinesiology, Sport, and Recreation, University of Alberta, AB T6G 2R3 Edmonton, Canada; 3 Department of Rehabilitation Medicine Research School CAPHRI, Maastricht University, 6200 MD Maastricht, The Netherlands; 4 Department of Radiology, Lithuanian University of Health Sciences, 44307 Kaunas, Lithuania; 5 Department of Imaging and Pathology, Group Biomedical Sciences, Biomedical MRI Unit, Catholic University Leuven, 3000 Leuven, Belgium; 6 Movement Control & Neuroplasticity Research Group, Group Biomedical Sciences, Catholic University Leuven, 3001 Heverlee, Belgium; 7 Department of Rehabilitation, Physical and Sports Medicine, Faculty of Medicine, Institute of Health Science, Vilnius University, 03101 Vilnius, Lithuania

**Keywords:** Neurogenesis, Sarcopenia, Strength training, Aging, Brain metabolism, N-acetylaspartate, Glutamate

## Abstract

Physical exercise is considered a potent countermeasure against various age-associated physiological deterioration processes. We therefore assessed the effect of 12 weeks of resistance training on brain metabolism in older adults (age range: 60–80 years). Participants either underwent two times weekly resistance training program which consisted of four lower body exercises performed for 3 sets of 6–10 repetitions at 70–85% of 1 repetition maximum (*n* = 20) or served as the passive control group (*n* = 21). The study used proton magnetic resonance spectroscopy to quantify the ratio of total N-acetyl aspartate, total choline, glutamate-glutamine complex, and myo-inositol relative to total creatine (tNAA/tCr, tCho/tCr, Glx/tCr, and mIns/tCr respectively) in the hippocampus (HPC), sensorimotor (SM1), and prefrontal (dlPFC) cortices. The peak torque (PT at 60°/s) of knee extension and flexion was assessed using an isokinetic dynamometer. We used repeated measures time × group ANOVA to assess time and group differences and correlation coefficient analyses to examine the pre-to-post change (∆) associations between PT and neurometabolite variables. The control group showed significant declines in tNAA/tCr and Glx/tCr of SM1, and tNAA/tCr of dlPFC after 12 weeks, which were not seen in the experimental group. A significant positive correlation was found between ∆PT knee extension and ∆SM1 Glx/tCr, ∆dlPFC Glx/tCr and between ∆PT knee flexion and ∆dlPFC mIns/tCr in the experimental group. Overall, findings suggest that resistance training seems to elicit alterations in various neurometabolites that correspond to exercise-induced “preservation” of brain health, while simultaneously having its beneficial effect on augmenting muscle functional characteristics in older adults.

**Supplementary Information:**

The online version contains supplementary material available at 10.1007/s11357-023-00732-6.

## Introduction

Aging is linked to numerous detrimental physical and functional changes in the human body [1–6]. The most common features of the aging process include the loss of muscle mass, strength and muscle function [1], brain structural and functional degradation along with cognitive decline [2, 3], altered brain metabolism [5, 7, 8], and other age-associated physiological changes such as elevated systemic inflammation [4]. The negative effect of age on muscle structure includes atrophy of type I and II myosin heavy chain isoforms [9], and decline in functional properties such as rate of force development, power, and maximal strength [6, 9]. These affected muscular characteristics can be primarily attributed to sarcopenia, an age-related loss in skeletal muscle mass and strength, accompanied by a decline in physical performance [6, 10]. Apart from sarcopenia-related muscular alterations, older adults’ physical and cognitive functioning are also affected by neurodegenerative processes [11–14]. For example, studies have shown that the hippocampus and the frontal lobe are significantly affected by age-related neuronal loss and the shrinking of gray matter [13]. In addition, recent evidence has indicated the interaction between sarcopenia and brain atrophy in regions such as the parietal lobe [14, 15]. The aforementioned age-dependent physical and functional changes in older adults thus culminate into several liability factors such as increased healthcare costs, old-age dependency ratio, and the need for more specialist physicians, all impacting the society [16]. Of interest, recent studies have suggested exercise-based intervention strategies for older adults, which can mitigate or even reverse to some extent these age-associated deteriorations of neuromuscular structures and neuronal loss in the brain [10, 17, 18].

An ideal measure that is occasionally recommended is the implementation of physical exercise like resistance training because of its potential counteracting effect on age-related deterioration of cognitive and motor function [19, 20]. Resistance training seems to be a perfect tool to enhance muscle strength in the elderly population and even preserve muscle mass against sarcopenia [10]. In addition, resistance training exhibits positive effects on functional changes in the brain and may prevent structural atrophy [21]. There is an increase in evidence suggesting a link between muscular strength and cognitive function in older adults [20, 21]. It was reported by Chen et al. that higher quadriceps isokinetic strength was associated with better executive function in the elderly population. Mavros et al. revealed that improvements in cognitive performance were mediated by the gains in the muscular strength attained by progressive resistance training in older adults with mild cognitive impairment [22]. Evidence has also shown the positive effects of physical exercise on inducing structural changes in the aging brain [18, 23, 24]. Colcombe and colleagues (2006) initially reported the beneficial effect of 6 months of aerobic training on increasing brain gray and white matter volume, particularly in prefrontal and temporal cortices regions [23]. An increase in hippocampal volume along with functional improvements in older adults was also observed after participating in 1 year of moderate-intensity aerobic exercise intervention [18].

Besides the prevention of brain tissue loss and cognitive function, physical exercise interventions also seem to affect neurometabolite concentrations in white and gray matter, which can be measured with proton magnetic resonance spectroscopy (^1^H-MRS). For example, aerobic training has been shown to increase relative N-acetyl aspartate (NAA) concentrations in the brain, primarily in the hippocampus and frontal gray matter, indicating exercise promotes neuronal integrity in these brain regions [25–27]. NAA is a neurometabolite which is highly expressed in cell bodies of neurons and could serve as a marker of brain neuronal integrity, neuronal density, or myelin synthesis [28, 29]. In normal aging, the NAA concentration and NAA to creatine ratio (NAA/Cr) have been reported to significantly decline in the frontal, temporal, and hippocampal regions of older adults [29]. In medical conditions like stroke, tumors, multiple sclerosis, and other neurodegenerative conditions like Alzheimer disease, a regional decline in NAA concentrations has also been observed [30]. Another amino acid that can be quantified by ^1^H-MRS is glutamate + glutamine (Glx), which is the combination of spectral peaks of glutamate and glutamine grouped together [28, 31]. Glutamate is the most abundant excitatory neurotransmitter in the human brain and is considered to be crucial in functional tasks like learning and memory due to its major role in synaptic information transmission [32, 33]. Similar to changes in NAA concentration, there is a decrease in the relative concentration of Glx in response to aging and cognitive impairment [34, 35]. Both acute (single bout) and chronic (multiple bouts over a period) aerobic exercise have been shown to induce an elevation of Glx levels in the brain [36–38]. Besides the neurometabolites like NAA and Glx, there is an age-dependent increase in neurochemical markers such as total choline (tCho; marker of membrane turnover), and myo-inositol (mIns; marker of glial cell activation) [29, 34, 39]. There have been mixed interpretations for tCho, as it is also viewed as a marker of phospholipid membrane synthesis and is observed in higher concentrations in aerobic exercise-trained individuals [26]. However, a concomitant increase in levels of tCho along with mIns is considered a marker of neuroinflammation and glial proliferation [40–42]. Overall, ^1^H-MRS seems to be a valuable tool to study the extent to which physical exercise can alter chemical concentrations of various neurometabolites in the human brain, allowing a deeper understanding of the mechanism behind the beneficial effects of physical exercise on the brain in terms of neural integrity, neuroinflammation, and neurodegeneration.

So far, studies investigating the effect of resistance training on brain health and exercise-induced neuroplasticity are limited compared to the number of studies investigating the effect of aerobic exercise [17]. Specifically, the effect of aerobic exercise on brain metabolism [25–27], changes in macromolecule levels like lactate [36], and alteration in other macromolecules such as intramyocellular lipids in skeletal muscle [43] have been intensively examined. However, to the best of the authors’ knowledge, there is a lack of evidence regarding the influence of resistance training on the localized neurochemical alterations in the brain. The current study aimed to explore the effects of 12 weeks of resistance training on the ^1^H-MRS-derived relative (creatine-referenced) levels of NAA, Glx, Cho, and mIns in three brain regions, i.e., the hippocampus, primary sensorimotor cortex, and pre-frontal cortex. We also aimed to examine if these potential changes in neurometabolite levels are associated with training-induced changes in peak torque of knee extension and flexion. Therefore, we hypothesized that an increase in peak torque in response to 12-week resistance training program would initiate effective neural developments in these brain regions, reflected by an increase in markers such as NAA/Cr and Glx/Cr ratios. Although there is lack of evidence on the effects of exercise on neurometabolites such as mIns and Cho, we speculated that after 12 weeks of resistance training, there would be a decrease in their levels, due to the anti-inflammatory nature of physical exercise [44].

## Methods

### Participants and study design

Forty-one (18 males and 23 females) older adults of age group 60–80 years, residing in Lithuania participated in the study. The participants were from the same pool of participants as mentioned in a previously published study by Vints et al. [42]. Patients with central nervous system (CNS) injuries, alcohol abuse, diabetes, musculoskeletal disorders, neurodegenerative diseases, or cancer were excluded from the study (see supplementary Table S1 for inclusion and exclusion criteria). A global cognitive assessment test (i.e., the Montreal Cognitive Assessment — MoCA) was administered to all participants by a qualified mental health care specialist (co-author SK). The MoCA test is considered a reliable and sensitive screening tool for identifying the risk of mild cognitive impairment (MCI) among older adults [45, 46] and was used in the present study to screen out dementia. The experimental protocol (supplementary Fig. 1) was sanctioned by the Kaunas Regional Biomedical Research Ethics Committee (No. BE-10–7), and a written informed consent was obtained from all participants prior to their inclusion process. This study was a 12-week randomized controlled trial which followed the stratified permuted block randomization procedure. The participants were stratified into subgroups of four based on a MoCA cutoff score of 26. Subsequently, permuted block randomization was used in each stratum, such that two participants each were randomly allocated to the experimental and control group. This ensured that both groups equally had two participants having a MoCA score of less than 26 while the other two participants having a MoCA score of 26 or higher. Investigators in data collection procedures were not involved in the intervention protocol in any form and as such were blinded to the group allocation, and similarly, the intervention administrators did not engage in the data collection process.

### Study protocol

All testing was done at the same time in the mornings, i.e., between 9 and 11 AM for all the participants. All the measurements were carried out in the Institute of Sport Science and Innovations, Lithuanian Sports University, Kaunas, within the same day, except for the ^1^H-MRS scanning sessions. These sessions were done in the Department of Radiology, Kauno Klinikos, Lithuanian University of Health Sciences, Kaunas, the previous day. For the testing days, the participants were instructed not to be involved in any vigorous activity at least two days prior to these appointments and to avoid drinking coffee on the test day. After arriving at the laboratory, participants were guided to sit calmly and quietly for 15 min. Following this procedure, all the participants completed the International Physical Activity Questionnaire-Short Form (IPAQ-SF). Total kcal/week was calculated based on their self-reported time spent on physical activity (PA) during a week. Total kcal/week burned during exercise was equal to total days of light/moderate/vigorous intense exercise in a week × average time performing light/moderate/vigorous intense exercise × METs, where METs (kcal/(kg × hour)) is the metabolic equivalent of task and is equal to 3.3/4.0/8.0 for light/moderate/vigorous intense exercise respectively [47, 48]. None of the recruited participants had prior experience with resistance-based training. Participants’ anthropometric measurements were taken after they completed the IPAQ-SF questionnaire. Height was measured using a standard stadiometer (cm), while body composition measures including weight (kg), body mass index (BMI, kg/m^2^), fat% and fat-free mass (FFM, kg) were measured using a Total Body Composition Analyzer (TBF-300A, Tanita Corporation). After the completion of anthropometric measurements, the participants’ handgrip strength (kg) was measured using a dynamometer (T.K.K.5401, Takei Digital Grip Strength Dynamometer). A 15-min interval was provided and the participants were directed to the final station of the isokinetic dynamometer for the strength measurements of knee extension and knee flexion movements.

### Brain imaging and^1^H-MRS

**Fig. 1:**
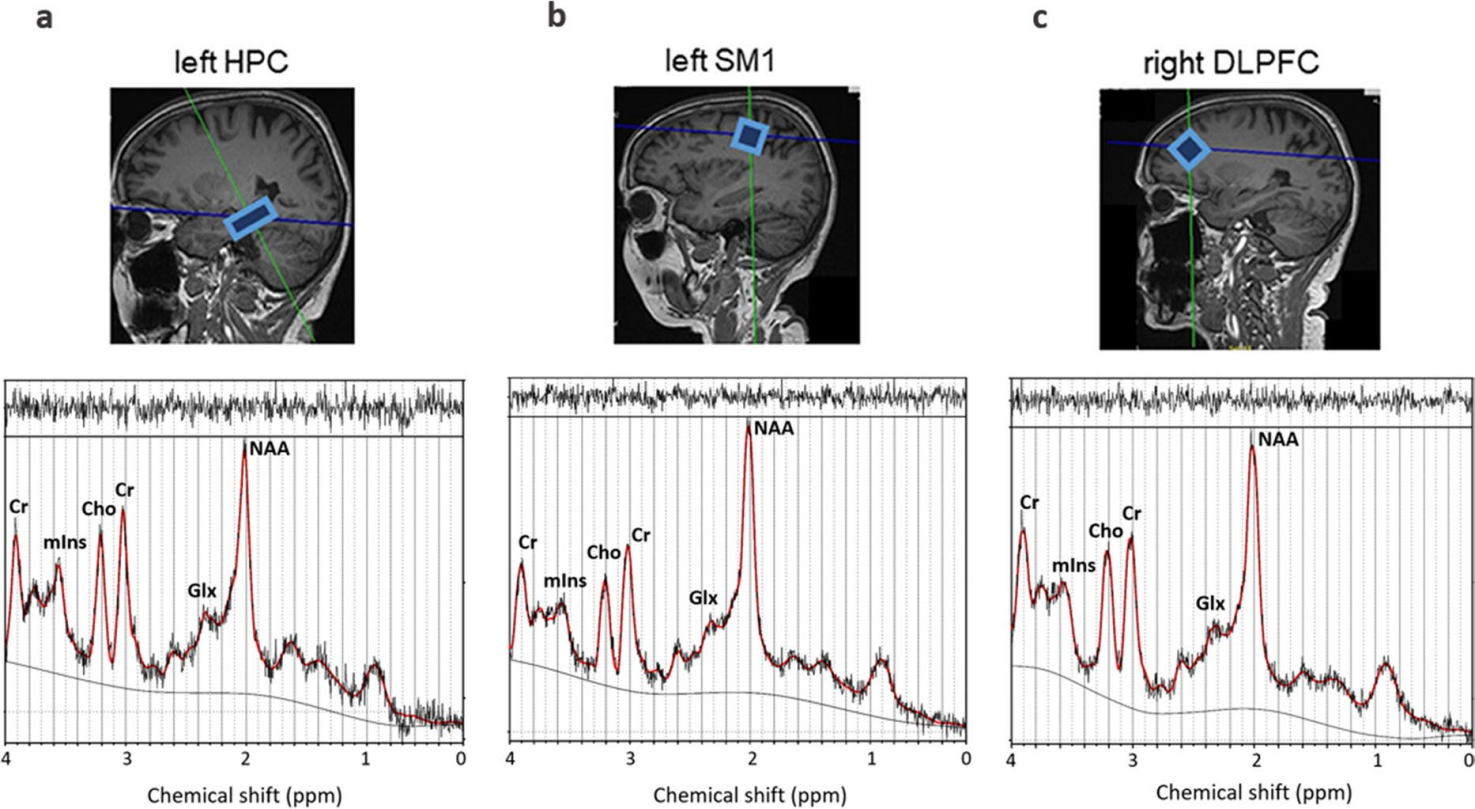
Example of voxel placement along with processed MR spectra in **a **the left hippocampus (HPC), **b** the left primary sensorimotor cortex (SM1), and **c** the right dorsolateral prefrontal cortex (dlPFC) in one participant

The brain MR imaging and ^1^H-MR spectroscopy were implemented as described in the study by Vints et al. 2022 [42]. All brain MR imaging was done using a Siemens 3 T Skyra MRI scanner (Siemens Healthineers, Erlangen, Germany) with a 32-channel receiver head coil. A high-resolution T1-weighted structural MR image (repetition time (TR) = 2200 ms, echo time (TE) = 2.48 ms, 0.9 × 0.9 × 1.0 mm^3^ voxels, field of view: 230 × 256 mm, number of sagittal slices = 176) was used to acquire a 3D magnetization prepared gradient echo (MPRAGE). MRS data were acquired using Point RESolved Spectroscopy (PRESS) sequence (TR = 2000 ms, TE = 30 ms, number of averages = 128, spectral bandwidth = 2000 Hz, data size = 1024 points) with excitation water suppression (sequence svs_se_30). The regions of interest (ROI) in which the ^1^H-MRS spectra were acquired included three voxel locations, namely the left hippocampus (HPC), left sensorimotor cortex (SM1), and right dorsolateral prefrontal cortex (dlPFC) (Fig. 1). The voxel sizes were as follows: (i) 1.6 × 1.6 × 1.6 cm^3^ in the SM1 and dlPFC voxels, and (ii) 26 × 12 × 12 cm^3^ in the HPC. The dlPFC and HPC were selected as ROIs, based on the evidence that these regions are considered the primary areas of brain plasticity following physical exercise [18, 49, 50], while SM1 acts as one of the fundamental cortices primarily involved with motor control and movement execution [51, 52].

MR spectra were processed using the totally automatic robust quantification through the linear combination of model spectra software (LCModel, version 6.3.1-R) [53]. Visual inspection was done to ensure there were no artifacts prior to quantification in LCModel. Quantified neurometabolites for all three voxels were (1) total NAA (tNAA) composed of N-acetyl aspartate and N-acetyl glutamate, (2) total Cr (tCr) composed of creatine and phosphocreatine, (3) total Cho (tCho) composed of phosphorylcholine and glycerophosphocholine, (4) mIns, and (5) glutamate–glutamine complex (Glx). The spectra needed to follow quality criteria before being included in statistical analysis, namely linewidths had to be less than 0.1 ppm and signal-to-noise ratio had to be greater than 5. All included neurometabolites were quantified with a Cramér-Rao lower bound (CRLB) < 20%. Water-referenced levels of tNAA, tCr, tCho, mIns, and Glx were quantified for each voxel location and ratios relative to tCr were calculated.

### Muscular strength measurements

The peak torque (PT) in knee extension and flexion was measured using an isokinetic Biodex System 3 dynamometer (Biodex Medical Systems, NY, USA). Prior to the testing protocol, the participants performed a 5-min warm-up by pedaling on a veloergometer, at a moderate intensity of 60–90 W corresponding approximately to their own body weight. This was followed by 3 min of dynamic activation exercises such as lunges, butt kicks, side step lunges, half-squats, and front and side cross swings. Only the dominant leg’s PT (right in all cases) for quadriceps (knee extension) and hamstrings (knee flexion) muscle groups were measured. The angular velocity was set at 60°/s and 3 repetitions at maximum intensity were performed. The highest PT (N.m) value attained during these 3 consecutive repetitions of knee extension and flexion was manually extracted for each participant for further analysis. The entire peak torque testing protocol was exactly repeated for all participants after 12 weeks.

### Resistance training intervention

The exercise intervention protocol was formulated according to the latest position statement by the National Strength and Conditioning Association (USA) for resistance training for older adults [54]. The intervention consisted of 12 weeks of progressive resistance training, with two training sessions per week and under the direct supervision of professionally qualified fitness instructors. The instructor-to-participant ratio was kept at 1:2 to ensure effective monitoring for all participants. Each training session started with a warm-up of 5-min cycling on a veloergometer, at an intensity (in Watts) approximately equaling to the participants’ body weight in kilograms. This was followed by a few dynamic stretching and activation exercises similar to as implemented during isokinetic strength testing. The exercise protocol consisted of four lower limb exercises.:(1) leg extension, (2) leg curl, (3) leg press, and (4) calf raises. It has been observed that lower limbs are more susceptible to aging-related loss of myofibers while arm muscles may remain largely unaffected [55]. Moreover, the physical function efficiency in the elderly is significantly dependent on their lower extremity muscle strength and power which ultimately correlates with the overall well-being of these individuals [56]. Therefore, this study implemented a lower-limb-focused resistance training program, and simultaneously, we expected that training of these large muscle groups may also increase the overall expression and production of myokines and angiogenesis factors [57, 58] that increase the probability of inducing exercise-induced neuroplasticity [59]. The two exercises (leg extension and leg curl) were the same exercises and movement patterns that were used during isokinetic peak torque calculations of the knee joint. A familiarization week consisting of two training sessions was conducted to teach the correct movement execution as well as to estimate the 1-repetition maximum (RM) of each individual for all the four exercises. The 1-RM assessment consisted of standard procedures of 1-RM testing protocol as outlined by the National Strength and Conditioning Association [60]. The predicted 1-RM was based on the number of repetitions performed at submaximal loads and calculating the 1-RM through the ExRx.net calculator (https://exrx.net/Calculators/OneRepMax) [61]. A total of three working sets and one warm-up set prior to the working sets were performed for all the four exercises. The inter-set rest intervals were 2 min long while the inter-exercise rest intervals were 3 min long. The intensity was kept between 70 and 85% of 1-RM for all working sets and exercises, and the repetition range was 6–10 [54]. Specifically, the participants exercised 1st to 3rd–week block at higher repetition ranges of 10 to 8 at 70–75% of 1-RM, followed by 4th to 9th–week block with an 8 to 6 repetition range at 75–80% of 1-RM and finally 10th to 12th–week block with 6 reps at 80–85% of 1-RM. Within these three blocks, the intensity was adjusted based on their rate of perceived exertion (RPE), quantified using the Borg-CR10 scale [62]. A target RPE of 7 or 8 on a 10-point scale was controlled within these blocks and the entire 12-week protocol. The participants had access to handouts of the RPE scale as a visual aid from the beginning of familiarization week. They were also consistently supported by the instructors to guide them with the rating on the Borg scale after each set. The instructors also maintained a training log throughout the intervention to note down the sessions’ repetition volume, exercise intensity, and RPE for each participant. The order of the exercises was not controlled; however, the sessions followed the principle of commencing from a multi-joint (leg press) exercise and finishing with single-joint (calf raises) exercises.

### Statistical analysis

All statistical analyses were performed with IBM SPSS Statistics (Version: 22.0, IBM Corp, 2020). The level of significance was set at *p* < 0.05. A time × group (*n*) repeated measure analysis of variances (ANOVA), with time (PRE and POST) as a within-subject factor and group (*n* = 2, specifically experimental and control) as a between-subject factor, was conducted to examine differences in PT of knee extension and knee flexion. A similar time × group (*n*) repeated measures ANOVA was applied for each of the three ROIs separately with four neurometabolite ratios (tNAA/tCr, tCho/tCr, mIns/tCr, and Glx/tCr) as four measures within each ANOVA. Post-hoc subgroup analysis tests were carried out if there were any significant time, group, or interaction effect observed from ANOVA results. The post-hoc test results were Bonferroni-adjusted for multiple comparisons. Upon visual inspection of the PT data, exploratory time × group (*n*) repeated measure ANOVA test was also performed to test the time factor (PRE and POST) and between-subject factor of group (*n* = 3 based on responders, non-responders, and control group). Data visualization was done by estimation plots generated using Data Analysis with Bootstrap-coupled ESTimation (DABEST, v0.3.1). A bootstrap 95% confidence interval (95% CI), which is bias-corrected and accelerated, was displayed by the estimation plots. The estimation plots are considered a robust method of data visualization that provides all the statistical information and avoids any data being concealed [63]. Further baseline exploratory analysis for responders and non-responders was done using estimation statistics: two-group Gardner-Altman plots and permutation *t*-tests.

Intervention-induced changes in peak torques and neurometabolite ratios were calculated as the percentage of pre-to-post differences relative to the pre measures: i.e., (∆) = 100 × [(Post − Pre) / Pre]. Positive values of ∆ indicated an increase in peak torque or an increase in neurometabolite ratio compared to baseline levels. Finally, bivariate correlation analyses (Pearson’s correlation) were performed between changes in PT (∆PT) with changes in neurometabolites’ ratio (∆tNAA/tCr, ∆tCho/tCr, ∆mIns/tCr, and ∆Glx/tCr) for both experimental and control group. Data visualization for the bivariate correlation analyses was done by plotting regression plots from Python seaborn library (Python, v3.9.12). A correction for multiple comparisons by false discovery rate (FDR) analysis (Benjamini and Hochberg adjustment) was carried out for all correlation analyses. In the FDR correction, each *p*-value is compared against a step-wise weighted critical value, to sequentially reject the null hypothesis starting from the smallest *p*-value to the largest. For exploratory analysis based on responders and non-responders, similar bivariate correlation analyses (Spearman's rank correlation due to the small sample size) were performed, followed by the FDR correction procedure.

## Results

### Participants’ data quality and baseline characteristics

Out of a total pool of 74 participants at baseline, 68 underwent 1H-MRS scanning and 65 had muscular strength measurements. The missing data in 1H-MRS dataset was a result of using different 1H-MRS scanning sequences of stimulated echo acquisition mode (STEAM) for a pilot study (*n* = 2), and mid-scanning termination due to uneasiness, claustrophobia, or discomfort (*n* = 4). One participant was excluded due to a pathology diagnosed during their brain imaging. In the strength measures, the missing data was due to the pilot inclusion (*n* = 2), fear of aggravating the knee-associated pain or other symptoms (*n* = 3), and initial unsuccessful storage of measurement data in the dynamometer-fed computer (*n* = 5). However, at follow-up, there were a high number of drop-outs (*n* = 23), which resulted in 42 participants completing the 1H-MRS scanning and 41 subjects having strength measures evaluation. The primary reasons for drop-out included incidences of multiple COVID-19 cases, lack of motivation for adherence, minor traumas, fear of injuries/training weights, and fear of getting infected during the pandemic. Overall, the dataset from 41 participants having experimental (*n* = 20) and control (*n* = 21) group was used for our present study (see Supplementary Table S2).

Participants’ baseline characteristics are described in Table 1. According to the IPAQ-SQ questionnaire, 80% and 95% of total participants within the experimental and control group respectively were moderate to highly active individuals. Based on the *t*-test results, no significant differences were found between the participants of the experimental and control group in their age, anthropometric characteristics, self-reported PA levels, and MoCA, suggesting that the two groups were homogeneous. The strength levels at baseline were similar in the experimental and control group, in handgrip strength, and PT of knee extension as well as knee flexion test measures. Finally, there were no baseline differences in neurometabolite ratios between groups in any of the three brain regions.

**Table 1:** Baseline group means (SD) of participants’ age, anthropometric characteristics, PA levels, MoCA score, handgrip strength, PT knee extension and flexion (60°/s)

Variable	Total (*n* = 41)	Experimental group (*n* = 20)	Control group (*n* = 21)	*p*-value (*t*-test)	Missing (*n*)
Age (years)	69.65 (5.57)	70.15 (5.33)	69.19 (5.85)	0.587	0
Height (cm)	166.47 (7.54)	165.30 (7.65)	167.65 (7.42)	0.331	1
Weight (kg)	76.64 (13.11)	78.07 (15.21)	75.22 (10.82)	0.499	1
BMI (kg/m^2^)	27.58 (3.86)	28.45 (4.30)	26.72 (3.25)	0.162	1
Fat (%)	32.42 (7.64)	34.08 (6.73)	30.85 (8.27)	0.191	2
FFM (kg)	51.85 (9.76)	51.75 (10.31)	51.96 (9.47)	0.948	2
PA levels (kcals/week)	3884.72 (3192.31)	3324.32 (3009.01)	4418.42 (3341.71)	0.278	0
MoCA score	25.22 (3.07)	25.60 (2.37)	24.85 (3.67)	0.448	1
Handgrip strength (kg)	31.93 (8.92)	32.16 (8.88)	31.73 (9.16)	0.884	1
PT knee extension (N.m)	117.45 (32.60)	117.55 (34.34)	117.36 (31.70)	0.985	0
PT knee flexion (N.m)	61.93 (15.77)	61.57 (16.72)	62.27 (15.23)	0.889	0

Significant at *p < 0.05 level. Abbreviations: *PT*, peak torque; *BMI*, body mass index; *FFM*, fat-free mass; *PA*, physical activity; *MoCA*, Montreal Cognitive Assessment. For the full dataset see Supplementary Table S3

### Resistance training induces changes in thigh muscle peak torque

**Fig. 2:**
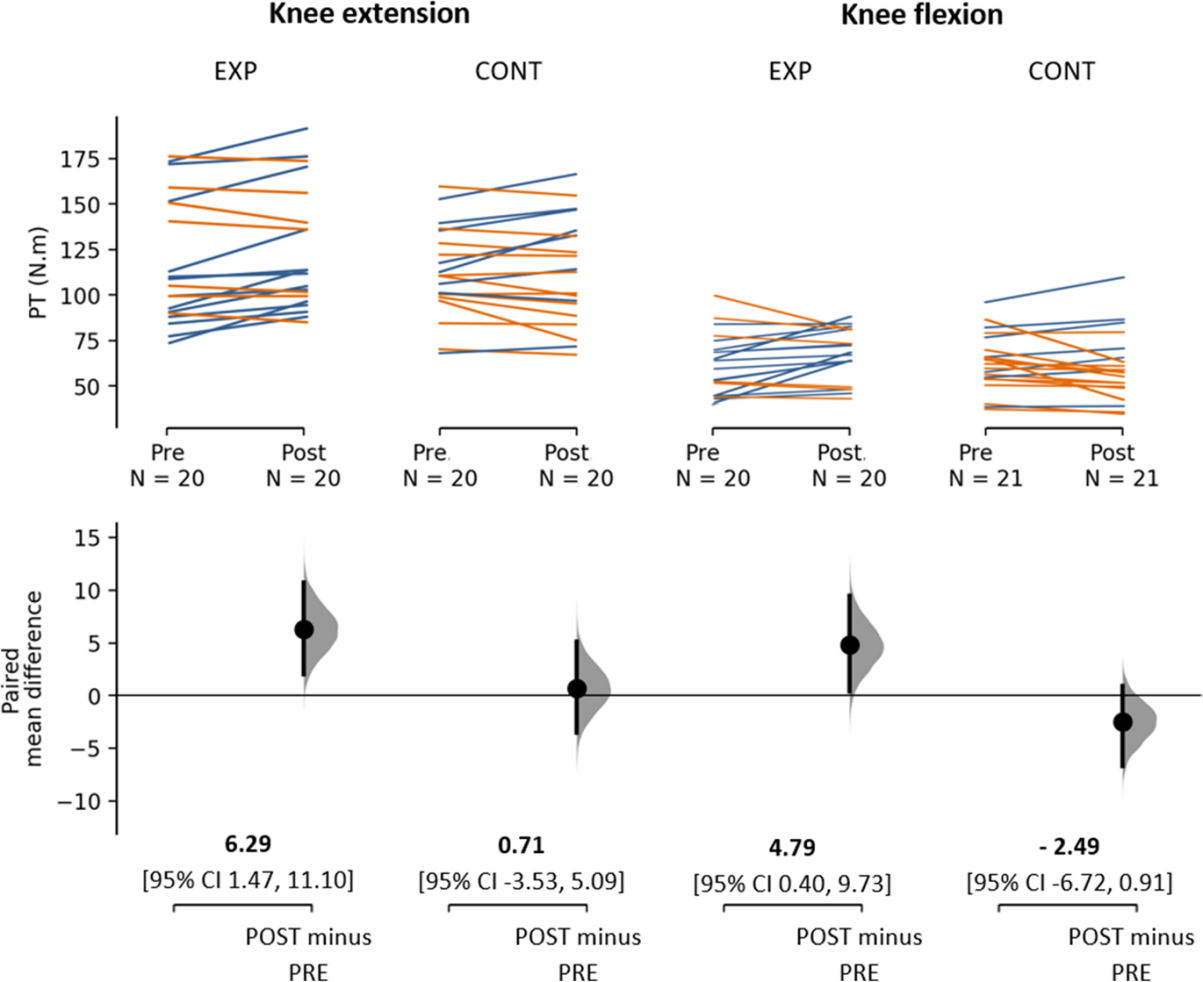
The paired mean difference for PT in EXP and CONT group are shown in the above Cumming estimation plot. The raw data is plotted on the upper axes; each paired set of observations for each participant from PRE to POST is connected by a line. Blue lines indicate POST minus PRE > 0 whereas orange lines indicate POST minus PRE < 0. On the lower axes, each paired mean difference is plotted as a bootstrap sampling distribution. Mean differences are depicted as dots in the Gaussian curve and 95% confidence intervals are indicated by the ends of the vertical error bars. The effect sizes and CIs are reported at the bottom as: mean difference [CI width lower bound, upper bound]. Abbreviations: PT, peak torque; EXP, experimental group; CONT, control group

The mean difference and pre-to-post changes of PT in knee extension and flexion are depicted in Fig. 2. Based on the results of time × group ANOVA, there was a significant interaction effect in PT knee flexion [F(1,39) = 5.665, *p* = 0.022, η_p_^2^ = 0.127] and a trend towards significant interaction effect with a moderate effect size in PT knee extension [F(1,39) = 3.112, *p* = 0.086, η_p_^2^ = 0.074]. Post-hoc subgroup analysis revealed a significant increase in PT knee flexion of the experimental group from pre-to-post (*p* = 0.035). In addition, there was a significant time effect for an increase in PT knee extension [F(1,39) = 4.942, *p* = 0.032, η_p_^2^ = 0.112]. Post-hoc tests indicated a significant increase in PT knee extension of the experimental group from pre-to-post (*p* = 0.008), but no changes in the control group (*p* > 0.200) (Supplementary Table S4).

Upon visual inspection, we observed that some of the participants in the experimental group showed a decrease in knee extension (*n* = 7, 35%) or flexion (*n* = 6, 30%) PT relative to their baseline values (Fig. 2). For the sake of additional exploratory analysis, participants within the experimental group were classified as responders and non-responders to the RT intervention. Responders (*n* = 8) were experimental group participants that exhibited an increase in PT knee extension of more than 5.09 N.m as well as an increase in PT knee flexion of more than 0.91 N.m, compared to baseline. These threshold values were set based on the upper bound value of 95%CI of the CONT group mean difference from pre-to-post in PT knee extension and flexion respectively. This ensured that the pre-to-post increase in PT of responders was not random effects. On the other hand, the remaining participants who did not clear any of the one threshold (either less than 5.09 N.m or 0.91 N.m) or had a decrease in any one of the PT of knee movement were classified as non-responders within the experimental group (*n* = 12). Results of time × group (3) ANOVA revealed a significant interaction effect in both PT knee extension and flexion [PT knee extension: F(2,38) = 13.628, *p* < 0.001, η_p_^2^ = 0.418; PT knee flexion: F(2,38) = 6.229, *p* = 0.005, η_p_^2^ = 0.247]. Post-hoc subgroup analysis revealed a statistically significant pre-to-post increase of PT knee extension (*p* < 0.001) as well as flexion (*p* = 0.018) in responders. No significant pre-to-post changes in PT measures were found in either non-responders or in control group participants (*p* > 0.200). There were no baseline differences among responders, non-responders, and control group as the main effect for group (3) was not significant [both PT knee extension and flexion: F ≤ 1.029, *p* > 0.300](Supplementary Table S5).

### Regional decline in tNAA/tCr and Glx/Cr after 12 weeks in the control group

A significant time effect in the dlPFC region for a decrease in tNAA/tCr and Glx/tCr was observed [tNAA/tCr: F(1,31) = 8.474, *p* = 0.007, η_p_^2^ = 0.215; Glx/tCr: F(1,31) = 4.517, *p* = 0.042, η_p_^2^ = 0.127]. Post-hoc subgroup analysis showed a significant decline with time in only dlPFC tNAA/tCr levels of control group participants (Table 2). A significant time effect for a decrease in Glx/tCr of SM1 region as well as a trend with moderate effect size of similar time effect in SM1 tNAA/tCr was observed [Glx/tCr: F(1,31) = 8.094, *p* = 0.008, η_p_^2^ = 0.207; tNAA/tCr: F(1,31) = 3.602, *p* = 0.067, η_p_^2^ = 0.104]. Post-hoc tests indicated a significant decline in SM1 Glx/tCr as well as tNAA/tCr in the control group. A significant time × group(2) interaction effect was seen in HPC Glx/tCr [F(1,29) = 5.929, *p* = 0.021, η_p_^2^ = 0.170]. However, post-hoc tests did not reveal any significant pre-to-post changes in the HPC region. There were no significant time, group, or interaction effect in tCho/tCr and mIns/tCr in any of the three regions [F < 2.5, *p* > 0.100] (supplementary Table S6).

**Table 2:** Group means (SD) along with mean difference (PRE to POST) of specific neurometabolite ratios in brain regions where ANOVA indicated significant time effects

Brain region	Group	PRE	POST	Mean difference [95% CI]	PRE-to-POST difference (%)
dlPFC tNAA/tCr	EXP	1.36 (0.11)	1.34 (0.11)	− 0.02 [95%CI − 0.07, 0.02]	− 1.65
	CONT	1.38 (0.10)	1.30 (0.11)	− **0.08 [95%CI** − **0.13,** − **0.02]**	− 5.52**
SM1 tNAA/tCr	EXP	1.55 (0.15)	1.53 (0.09)	− 0.02 [95%CI − 0.10, 0.06]	− 0.57
	CONT	1.62 (0.14)	1.53 (0.15)	− **0.09 [95%CI** − **0.17,** − **0.01]**	− 5.16*
SM1 Glx/tCr	EXP	1.05 (0.26)	0.96 (0.19)	− 0.08 [95%CI − 0.22, 0.05]	− 3.05
	CONT	1.08 (0.16)	0.89 (0.15)	− **0.19 [95%CI** − **0.33,** − **0.04]**	− 16.44*

Abbreviations: *dlPFC*, prefrontal cortex; *tNAA*, total N-acetyl aspartate; *tCr*, total creatine; *SM1*, sensorimotor cortex; *Glx*, glutamine-glutamate complex. Bold entries are significant at the *p<0.05 or **p<0.01 level

Additional exploratory analysis with sub-classification of participants in responders and non-responders did not reveal any significant time or interaction effect in any of the three regions (*p* > 0.100). However, there were significant group effects observed in tNAA/tCr and mIns/tCr of HPC region (see supplementary Table S7). Post hoc tests for multiple comparisons indicated that non-responders had significantly higher mIns/tCr compared to responders.

### Exploratory analysis to assess the differences between responders and non-responders within experimental group

**Fig. 3:**
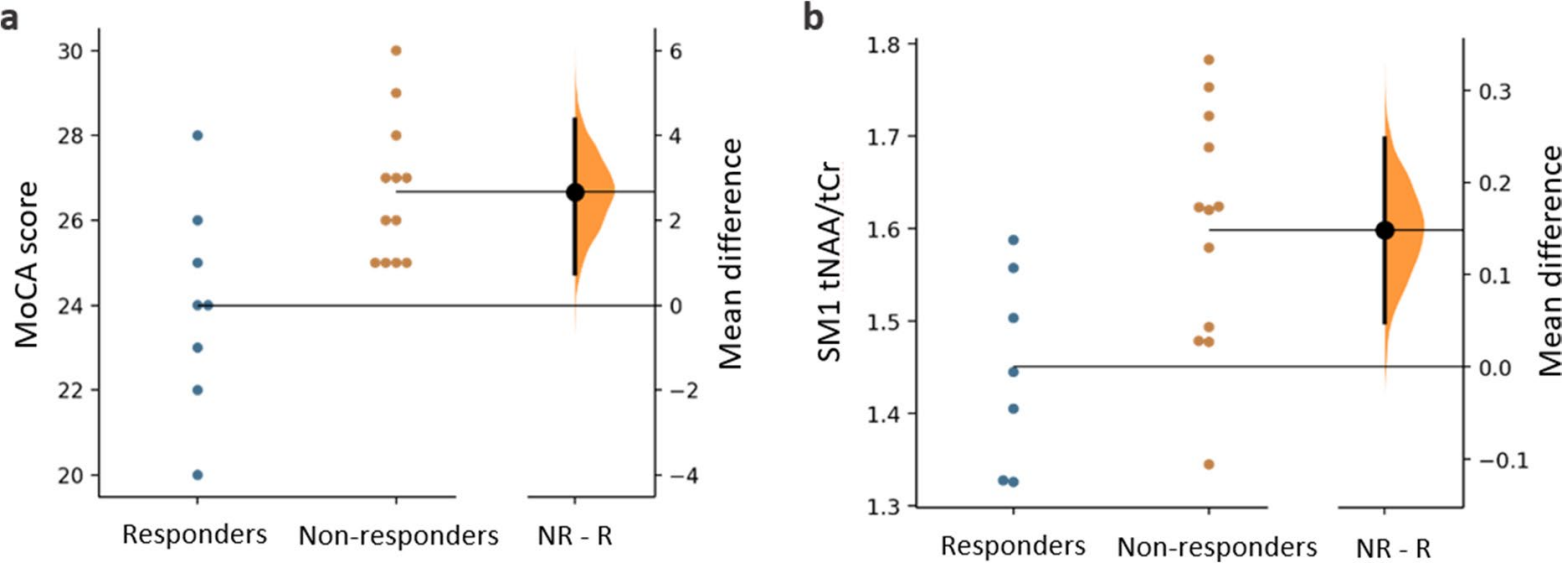
The mean differences between responders and non-responders at baseline is shown in the above Gardner-Altman estimation plots for **a** MoCA score (*n* = responders, 8; non-responders, 12) and (**b**) SM1 tNAA/tCr (*n* = responders, 7; non-responders, 12). Individual results for both groups are plotted on the left axes; the mean difference is plotted on a floating axes on the right as a bootstrap sampling distribution. The mean difference is depicted as a dot between the Gaussian curve and the 95% confidence interval is indicated by the ends of the vertical error bar. Abbreviations: MoCA, Montreal cognitive assessment; NR-R, mean difference between non-responders and responders; SM1 tNAA/tCr, sensorimotor cortex concentration ratio of total N-acetyl aspartate relative to total creatine

Exploratory permutation *t*-test results indicated that were no baseline differences between responders and non-responders in age and anthropometric characteristics including weight, BMI, FFM, or fat% (Table 3). Moreover, there were no differences in their self-reported PA levels based on the average energy expended during a week. However, in MoCA test scores, the non-responders had significantly higher MoCA performance as compared to responders at baseline. In particular, out of 8 total responders, 6 of them had a MoCA score of ≤ 25 indicating a high risk of MCI within this population. On the other hand, among the total of 12 non-responders, only 4 participants had a MoCA score of ≤ 25. In strength measures based on handgrip and PT tests, both groups had homogeneous strength levels although a statisitcally insignificant trend of higher baseline strength in non-responders compared to responders was seen. At the level of relative neurometabolite ratios in the SM1 region, there were baseline differences in tNAA/tCr between responders and non-responders. However, correcting for multiple comparisons in neurometabolite ratios, this difference in SM1 tNAA/tCr became insignificant. The results of two-independent-groups mean difference estimation plots for MoCA score and SM1 tNAA/tCr are presented in Fig. 3.

**Table 3:** Exploratory permutation *t*-test analysis to assess baseline differences between responders and non-responders

Test measure	Responders (*n* = 8)	Non-responders (*n* = 12)	Mean difference [95% CI lower bound, upper bound]	Permutation *t*-test *p*-value
Age (years)	70.37 (7.24)	70.00 (3.95)	− 0.37 [95% CI − 4.83, 5.46]	0.862
Weight (kg)	76.82 (11.90)	78.90 (17.53)	2.08 [95% CI − 11.1, 13.4]	0.778
BMI (kg/m^2^)	27.67 (4.03)	28.96 (4.57)	1.30 [95% CI − 2.07, 5.13]	0.525
Fat (%)	35.41 (8.52)	33.30 (5.73)	− 2.11 [95% CI − 7.18, 7.31]	0.530
FFM (kg)	50.68 (9.26)	52.37 (11.22)	1.69 [95% CI − 7.86, 10.0]	0.741
PA levels (kcals/week)	2423.93 (2423.43)	3924.58 (3304.38)	1500 [95% CI − 1060, 3770]	0.284
MoCA score	24.00 (2.44)	26.67 (1.66)	**2.67 [95% CI 0.75, 4.38]**	0.006**
Handgrip strength (kg)	30.42 (7.32)	33.16 (9.84)	2.74 [95% CI − 4.58, 10.10]	0.513
PT knee extension (N.m)	106.90 (36.52)	124.65 (32.42)	17.8 [95% CI − 14.10, 44.01]	0.268
PT knee flexion (N.m)	56.08 (13.46)	65.23 (18.19)	9.15 [95% CI − 3.65, 22.7]	0.239
HPC tNAA/tCr	1.13 (0.14)	1.22 (0.18)	0.09 [95% CI − 0.04, 0.25]	0.272
HPC tCho/tCr	0.31 (0.03)	0.32 (0.04)	0.01 [95% CI − 0.01, 0.05]	0.428
HPC Glx/tCr	1.69 (0.31)	1.78 (0.43)	0.09 [95% CI − 0.22, 0.42]	0.628
HPC mIns/tCr	0.95 (0.15)	1.10 (0.17)	0.15 [95% CI 0.01, 0.303]	0.083
SM1 tNAA/tCr	1.49 (0.16)	1.59 (0.13)	**0.15 [95% CI 0.04, 0.24]**	0.020*
SM1 tCho/tCr	0.26 (0.03)	0.28 (0.04)	0.01 [95% CI − 0.01, 0.05]	0.395
SM1 Glx/tCr	0.97 (0.17)	1.10 (0.30)	0.12 [95% CI − 0.05, 0.35]	0.331
SM1 mIns/tCr	0.68 (0.15)	0.71 (0.13)	0.02 [95% CI − 0.09, 0.15]	0.672
dlPFC tNAA/tCr	1.35 (0.09)	1.36 (0.12)	0.007 [95% CI − 0.09, 0.09	0.890
dlPFC tCho/tCr	0.31 (0.02)	0.31 (0.03)	0.008 [95% CI − 0.01, 0.03]	0.599
dlPFC Glx/tCr	1.18 (0.25)	1.27 (0.20)	0.08 [95% CI − 0.14, 0.25]	0.422
dlPFC mIns/tCr	0.81 (0.14)	0.84 (0.13)	0.02 [95% CI − 0.08, 0.15]	0.633

Permutation *t*-tests with mean difference [95% CI lower bound, upper bound] for all test measures within responders and non-responders. The results of neurometabolite ratios are presented prior to the FDR correction procedure. Abbreviations: *BMI*, body mass index; *FFM*, fat-free mass; *PA*, physical activity; *PT*, peak torque; *MoCA*, Montreal cognitive assessment; *dlPFC*, dorsolateral prefrontal cortex; *HPC*, hippocampal cortex; *SM1*, sensorimotor cortex; *Glx*, glutamine-glutamate complex; *mIns*, myoinositol; *tCho*; total choline; *tCr*, total creatine; *tNAA*, total N-acetyl aspartate. Bold entries are significant at the *p<0.05 or **p<0.01 level

### Association of resistance training-induced changes in muscle strength with changes in brain neurometabolite ratios

**Fig. 4:**
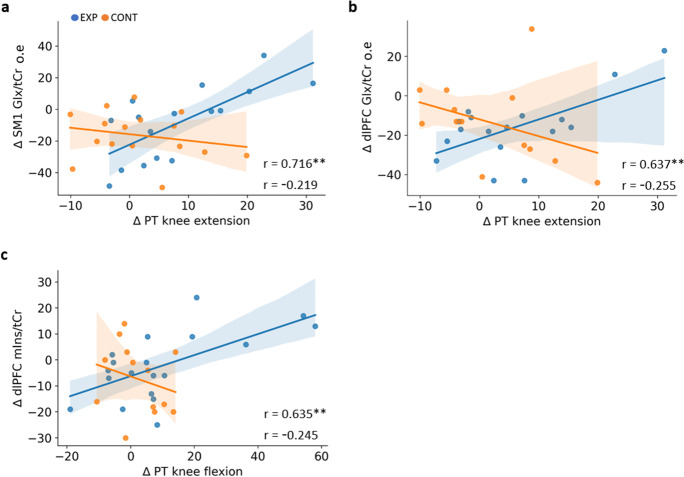
The linear relationship that survived FDR procedure between changes in strength and neurometabolite ratios were **a** ∆PT knee extension and ∆SM1 tNAA/tCr (*n* = EXP, 16; CONT, 16), **b** ∆PT knee extension and ∆dlPFC Glx/tCr (*n* = EXP, 17; CONT, 13), and **c** ∆PT knee flexion and ∆dlPFC mIns/tCr (*n* = EXP, 19; CONT, 14), in EXP (blue) and CONT (orange) group participants. See Supplementary Fig. 2 for all relationships. Abbreviations: SM1, sensorimotor cortex; Glx, glutamine-glutamate complex; tCr, total creatine; PT, peak torque; dlPFC, pre-frontal cortex; mIns, myo-inositol; EXP, experimental group; CONT, control group

The results of Pearson’s correlation indicated both ∆PT of knee extension and flexion were positively associated with ∆SM1 tNAA/tCr, ∆SM1 Glx/tCr, and ∆dlPFC Glx/tCr. Contrary to what we expected, there was also a positive correlation between ∆PT knee flexion and ∆dlPFC mIns/tCr. The correlation of ∆PT of knee extension with ∆SM1 tNAA/tCr did not survive the FDR correction for multiple testing, while with ∆SM1 Glx/tCr (*R*^2^ = 0.512 [95%CI 0.212, 0.813]) and ∆dlPFC Glx/tCr (*R*^2^ = 0.401 [95%CI 0.081, 0.720]) remained significant. The correlation between ∆PT knee flexion and ∆dlPFC mIns/tCr (*R*^2^ = 0.397 [95%CI 0.091, 0.703]) also remained significant after the FDR procedure (Fig. 4). In the control group, a positive relationship between ∆PT knee flexion and ∆HPC tCho/tCr as well as a negative relationship between ∆PT knee flexion and ∆dlPFC mIns/tCr was found; however, none of them survived FDR correction (see Supplementary Table S8 for all correlations). There were no other associations between ∆PT of knee movement and ∆brain neurometabolite ratios in control group participants. Among the neurometabolites, there was a positive correlation between ∆tNAA/tCr and ∆mIns/tCr in both HPC (*p* = 0.002) and dlPFC region (*p* = 0.012) of the experimental group. In addition, a positive association was found between ∆tNAA/tCr and ∆Glx/tCr in SM1 region (*p* = 0.004). None of these correlations between pre-to-post differences in neurometabolites survived the FDR correction for multiple testing (Supplementary Table S9).

Following the sub-classification of experimental group participants by peak torque gain, the responders showed a positive association between ∆PT knee extension and ∆dlPFC Glx/tCr that survived FDR correction (Table 4). A similar positive relationship was seen between their ∆PT knee flexion and ∆SM1 tNAA/tCr. The positive association of ∆PT knee flexion with ∆dlPFC mIns/tCr was also observed in responders as observed earlier in the entire experimental group. In addition, there was a positive correlation of ∆PT knee extension with ∆mIns/tCr in HPC as well as dlPFC. However, except for the correlation of ∆PT knee flexion with ∆dlPFC mIns/tCr, the latter two *p*-values did not survive the FDR correction for multiple testing. At baseline, there was a negative correlation between PT knee extension and SM1 mIns/Cr (*p* = 0.048), but it did not survive the FDR procedure. Apart from this association, experimental and control group participants did not show significant correlations between the initial strength levels and the neurometabolite ratios in HPC, SM1, and dlPFC (*p* > 0.100). Similarly, responders and non-responders did not exhibit any associations between baseline strength levels and neurometabolite ratios in any of the three regions (*p* > 0.100).

**Table 4:** The relationship between changes (∆) in PT knee extension/flexion after resistance training with changes (∆) in brain neurometabolite ratios among responders and non-responders

		Responders		Non-responders	
		Spearman’s *ρ*	*p*-value	Spearman’s *ρ*	*p*-value
∆ PT knee extension	∆ HPC tNAA/tCr	0.829	0.042*	0.409	0.212
	∆ HPC mIns/tCr	0.943	0.005**	0.273	0.417
	∆ SM1 tNAA/tCr	0.736	0.036*	0.152	0.676
	∆ dlPFC Glx/tCr o.e	0.943	0.005**	0.191	0.574
	∆ dlPFC mIns/tCr	0.821	0.023*	− 0.105	0.746
∆ PT knee flexion	∆ HPC Glx/tCr	− 0.029	0.957	0.736	0.010*
	∆ SM1 tNAA/tCr	0.964	0.000***	0.176	0.627
	∆ SM1 Glx/tCr o.e	0.857	0.014*	0.117	0.765
	∆ SM1 tCho/tCr	0.857	0.014*	− 0.164	0.651
	∆ dlPFC mIns/tCr	0.893	0.007**	0.476	0.118

Spearman’s rank correlation coefficient analysis for ∆ PT of knee movement. **p < 0.01; *p < 0.05. Only statistically significant correlations are presented (for full data set see Supplementary Table S10). Correlations significant after FDR procedure are highlighted. Abbreviations: *PT*, peak torque; *dlPFC*, dorsolateral prefrontal cortex; *HPC*, hippocampal cortex; *SM1*, sensorimotor cortex; *o.e.*, influential outlier excluded; *Glx*, glutamine-glutamate complex; *mIns*, myoinositol; *tCho*, total choline; *tCr*, total creatine, *tNAA*, total N-acetyl aspartate

## Discussion

Our study aimed to investigate the resistance training–induced alterations in brain metabolism of older adults in the hippocampus, primary sensorimotor cortex, and prefrontal cortex. We hypothesized that 12 weeks of resistance training would be associated with the increased relative concentration of tNAA and Glx, which may indicate increased neuronal density and enhanced glutamatergic neurotransmission in these particular brain regions. Although, there were no evident increases in tNAA and Glx after resistance training, relatively stable measures of tNAA/tCr and Glx/tCr were observed in the experimental group after 12 weeks. On the contrary, the control group experienced a significant decline in tNAA/tCr of SM1 and dlPFC cortices. Along with the reductions in tNAA/tCr, there was a similar decrease in Glx/tCr of the SM1 region in control group participants following 12 weeks. These results are in agreement with cross-sectional studies between young and old adults that have reported an age-associated decrease in NAA and Glx in the prefrontal as well as sensorimotor cortices [5, 8, 29]. Although 12 weeks may be considered a relatively short duration to observe neurochemical alterations, limited evidence has reported a significant increase in tCho/tCr (marker of membrane turnover) within the same follow-up duration in passive control older adults, participating in an aerobic training-based randomized controlled trial [64]. In the present study, there was an average decline of 5.16% and 5.52% in tNAA/tCr of SM1 and dlPFC respectively in the control group, as opposed to a 0.57% and 1.65% decrease in the experimental group over 12 weeks. Longitudinal studies examining age-related NAA changes have reported contrasting results. For example, Sijens et al. found an annual decline of about 3.8% in NAA/Cr levels in older adults aged between 65 to 81 years [65], while other longitudinal studies have reported relatively stable NAA/Cr levels in older adults of similar age with time [66, 67]. Even though the tNAA/tCr decreases in our control population are larger than would be expected from previous literature, the observed concomitant decrease of tNAA/tCr and Glx/tCr in our study strengthens our interpretation that the changes most likely reflect an age-associated decrease in neuronal density. The loss of neurons could lead to impaired glutamatergic neurotransmission which explains the reduced Glx levels [68, 69]. The literature has also shown that structural atrophy specifically in prefrontal and sensorimotor regions is highly prevalent in the older population [70–72]. It is very likely that age-associated neurodegenerative processes along with changes in brain tissue may have influenced alterations of these neurometabolite ratios, or vice-versa. It is also interesting to note that based on our sub-classification of exercising older adults, responders even exhibited an average increase of 3.76% in SM1 tNAA/tCr while non-responders exhibited a decline of 3.61% after 12 weeks. Overall, these results substantiate the potential of resistance training in preserving against the aging-related brain deterioration in older adults as encountered by the non-responders and control group. This is somewhat in line with findings from aerobic exercise studies, which have found that hippocampal volume is preserved in the exercise group, while the control group showed an age-related volume loss [18, 73]. Following 12 weeks, no changes were seen in mIns or tCho relative to tCr, in any of the two groups. To our knowledge, there is no evidence regarding the effect of physical exercise on mIns levels. However, a few studies focusing on concussion and contact sports have observed increased mIns levels in the primary motor cortex suggesting neuroinflammation due to repeated exposure to head impacts [74, 75]. The absence of significant changes in mIns/Cr in our study may imply that neither our resistance training protocol induces a neuroinflammatory response in older adults, nor there are evident age-associated changes in neuroinflammatory levels of both group participants after 12 weeks.

Interestingly, we observed a positive association of pre-to-post gains (∆) in peak torque of knee extension and flexion with ∆tNAA/tCr, ∆Glx/tCr (SM1 region) as well as with ∆Glx/tCr and ∆mIns/tCr (dlPFC region) of the experimental group. Previous literature has reported positive effects on the relative concentration of NAA levels in the hippocampus after 3 months of aerobic exercise [49, 76]. In addition, some cross-sectional studies have reported higher aerobic fitness being associated with higher baseline NAA/Cr levels in the brain [25, 26]. Similarly, a few studies have seen an increase in Glx following acute [36] and chronic high-intensity aerobic exercise [37]. However, the present study is, to the best of our knowledge, the first to provide evidence that the extent of resistance training-induced effect on mediating brain metabolism in older adults seems to directly depend on their muscle strength gains. It should be noted, that only ∆PT associations with ∆Glx/tCr and ∆mIns/tCr survived the FDR procedure. The positive association between ∆PT and ∆Glx/tCr may possibly be due to the fact that muscular adaptations directly led to an increased overall tissue pool of glutamate [38] and/or altered the expression of glutamatergic receptors [77]. Moreover, this exercise-dependent increase in Glx may have also sequentially led to an increase in NAA, as seen by the positive association between SM1 ∆Glx/tCr and ∆tNAA/tCr prior to FDR procedure. This association can be backed up by the involvement of glutamate and glutamine in NAA synthesis through metabolic pathways such as the tricarboxylic acid cycle which have been well documented [78]. However, the regulatory mechanisms related to the exercise-dependent increase in the expression of Glx and NAA still remain speculative. Contradictory to what we would have expected, there was a positive association between ∆PT and ∆mIns/tCr in dlPFC region. This association suggests that extreme increases in muscle strength could potentially lead to increased expression of glial proliferation. Evidence shows mIns is primarily found in astrocytes and acts as a marker of astrocytic activity and neuroinflammation [79]. However, these astrocytes are specialized glial cells and have consistently been observed to be activated as well as proliferated by physical exercise [80–82]. Ekdahl et al. (2009) described the multifaceted role of these glial cells and their skeptical function in increasing the number of synapses, neural circuit function, brain plasticity, and neuroprotective effects [83]. It should also be noted that although we observed significant improvements in PT after 12 weeks of resistance training, there were no changes observed in mIns/tCr per se in any of the three brain regions. Therefore, it becomes clearer that resistance training in itself may not be stimulating neuroinflammation or enhancing astrocytic activity. Rather, this ∆PT and ∆mIns/Cr relationship could be indicative that muscular adaptations due to resistance training are positively associated with activating the indirect pathways of microglia-induced neurogenesis [82, 83]. This idea can be partly backed up by the finding that ∆tNAA/tCr and ∆mIns/tCr were also positively correlated with each other, in HPC as well as in the dlPFC before the FDR procedure. In line with all these observations and our findings, it is intriguing to suggest that resistance training, potentially through multiple pathways, can serve as an ideal tool to initiate concurrent improvements in the muscle and brain health of older adults.

It is also important to discuss the potential factors that might explain the strength gain phenotype we observed, in response to resistance exercise within the experimental group. Previous studies have suggested factors such as nutritional habits, genetic variations including individual genetic polymorphisms such as in ACTN3 gene, and baseline characteristics that can justify the variability in response to exercise among individuals [84–87]. In the present study, there were baseline differences in two measures between responders and non-responders of the experimental group. Specifically, responders demonstrated potential cognitive deficits with significantly lower MoCA scores and lower SM1 tNAA/tCr as opposed to non-responders at baseline. MoCA is one of the most widely used cognitive screening tools within health-care, proven to have a higher sensitivity as compared to the traditional Mini-Mental State Exam for the detection of cognitive impairment or dementia [45, 46]. An established cutoff score of 26 is commonly adhered to screen the risk of MCI in older adults [46]. However, recent meta-analyses have suggested lower MoCA cutoff scores of 23 [45] or 24 [88] to effectively screen for MCI and reduce rates of false positives. Even though it is beyond the scope of this study to strictly classify responders as MCI individuals based on their MoCA score, it is evident that irrespective of the cutoff being used, responders had slight cognitive impairment in comparison to non-responders, based on their worse MoCA test scores. Along with this indicator of potential brain functional impairment, responders in the present study also had significantly lower baseline tNAA/tCr in SM1 region prior to FDR procedure. Levin et al. showed that lower SM1 NAA levels correlated with worse bimanual coordination performance and lower fine motor ability among older adults. These reduced NAA levels could reflect impaired functional network integrity in SM1 which supports bimanual coordination domain of motor control [8]. Another study observed that participants that responded to 16 weeks of a multimodal exercise program had worse baseline motor performance than non-responders [89]. Taken together, lower SM1 tNAA/tCr in our study’s responders might be indicatory of their lower motor control integrity or other deficits which possibly made them more receptive to the motor task (i.e., resistance training intervention in our study). In addition, some studies have also shown a positive association between MoCA scores and NAA/Cr in mild cognitively impaired older adults [90, 91]. Therefore, lower tNAA/tCr in our responders can also be a potential underlying factor that led to the functional cognitive deficits, evidenced by their worse MoCA performance. In line with all evidence, it could be considered that the initial neuropsychological and neurochemical parameters of older adults within the experimental group might be some of the factors that determined the inter-individual differences in resistance training. Accordingly, it is important to consider that the non-responders were categorized based on the exercise intervention that was implemented in our study. Evidence suggests adjusting modalities such as the dose (exercise intensity, volume, duration, type) of resistance training intervention can mitigate this exercise inter-individual heterogeneity, as non-responders do respond to a higher dose of training (Montero & Lundby, 2017; Pickering & Kiely, 2019).

Overall, based on our findings, it can be concluded that resistance training may be protective against aging-associated neurodegeneration and altered neurometabolism in primary sensorimotor and prefrontal cortices. The beneficial effect of resistance training is not only localized to the specific muscles being trained but in addition could concurrently lead to enhanced brain neuronal health. The older adults who underwent resistance training had a significant increase in their muscle strength and at the same time experienced to some extent an exercise-dependent increase in neurochemical markers of brain neuronal density and enhanced neurotransmission. Considering the exploratory nature of this study, the results should be considered keeping into account certain limitations. The sample size was small and was further reduced as a result of the sub-classification within the experimental group based on exercise response. Secondly, the exact order of exercises was not controlled and could potentially affect the strength gains among the participants. Finally, it should be noted that we did not perform tissue correction for ^1^H-MRS-derived metabolites, but instead, neurometabolite concentration ratios relative to tCr were reported. This serves as an internal reference for neurometabolite levels and is much less likely to be influenced by contributions of cerebrospinal fluid [94]. Nevertheless, this study should encourage future research into further examining the effect of resistance training on markers of brain neuronal integrity, tissue volume, and outcome measures relating to older adults’ brain health. It would be enlightening to study the potential neuroprotective effects of resistance training in older adults through a longitudinal design to gain more insights into the alterations in brain metabolism over a period. In addition, it would also be informative for future studies to examine the role of potential biomarkers, called exerkines [59], that are considered to mediate the exercise-dependent muscle-brain crosstalk to better understand the mechanisms behind the training adaptations. Lastly, future studies may look into specific interventions by modifying different interventional variables of exercise dose, which could limit the amount of responders and non-responders within the interventional group.

## Supplementary Information

Below is the link to the electronic supplementary material.

Supplementary file1 (DOCX 833 KB)

## Data Availability

Excel files with processed data and statistical outputs supporting the conclusions of this article will be made available by the authors upon request, without undue reservation. Obtaining access to raw data or MRI scan files will require approval from the project manager (nerijus.masiulis@lsu.lt) in addition to ethical approval (on an individual user and purpose basis) by the local medical ethical committee. The authors are willing to support such ethical approval applications.
