# Supplementary material for: Strength gains after 12 weeks of resistance training correlate with neurochemical markers of brain health in older adults: a randomized control 1H-MRS study
Source: GeroScience. 2023 Jan 26;45(3):1837–55. doi: 10.1007/s11357-023-00732-6 (PMC9877502; doi:10.1007/s11357-023-00732-6)

**Strength gains after 12 weeks of resistance training correlate with neurochemical markers of brain health in older adults: a randomized control ^1^H-MRS study**

**Samrat Sheoran^1,2 *^, Wouter A.J. Vints^1,3^, Kristina Valatkevičienė^4^, Simona Kušleikienė^1^, Rymantė Gleiznienė^4^, Vida J. Česnaitienė^1^, Uwe Himmelreich^5^, Oron Levin^1,6^, Nerijus Masiulis^1,7^**

^1^Department of Health Promotion and Rehabilitation, Lithuanian Sports University,

LT-44221 Kaunas, Lithuania

^2^Faculty of Kinesiology, Sport, and Recreation, University of Alberta, Edmonton, Canada

^3^Department of Rehabilitation Medicine Research School CAPHRI, Maastricht University P.O. Box 616, 6200 MD Maastricht, The Netherlands

^4^Department of Radiology, Lithuanian University of Health Sciences, Kaunas, Lithuania

^5^Biomedical MRI Unit, Department of Imaging and Pathology, Group Biomedical Sciences, KU Leuven, Leuven 3000, Belgium

^6^Movement control & Neuroplasticity Research Group, Group Biomedical Sciences, KU Leuven, Heverlee 3001, Belgium

^7^Department of Rehabilitation, Physical and Sports Medicine, Institute of Health Science, Faculty of Medicine, Vilnius University, LT-03101 Vilnius, Lithuania

*Corresponding author:

Samrat Sheoran

Clinical and Theoretical Neurophysiology Lab

Van Vliet Complex, University of Alberta

Edmonton, Alberta T6G 2H9

Tel. +91 74475 20295

Email: ssheoran@ualberta.ca

| **Supplementary Table S1.** Exclusion and inclusion criteria and screening protocol for inclusion | |
| --- | --- |
| **Inclusion criteria** | **Exclusion criteria** |
| Aged ≥60 years | Diagnostic of dementia,  Montreal Cognitive Assessment (MoCA) ≤16 |
| Adequate visual, auditory, and fine motor skills | Consumption of psychopharmacological drugs current or during last 5 years; or more than 5 years throughout life |
| Fluent in Lithuanian | Diagnostic of neurological disorder: stroke, epilepsy, multiple sclerosis, traumatic brain injury, brain tumor, neurodegenerative disease |
| Acceptance of participation in the study and signature of the informed consent | Diagnostic of psychiatric illness current or during last 5 years |
|  | Diabetes |
|  | History of drug abuse or alcoholism current or during last 5 years; or more than 5 years throughout life |
|  | History of chemotherapy |
|  | Musculoskeletal disorders |
|  | Contraindication to magnetic resonance imaging |

| **Supplementary Table S2.** Total number of good quality measurements for PT and ^1^H-MRS at PRE and POST in both groups, n = 41. | | | | | | | |
| --- | --- | --- | --- | --- | --- | --- | --- |
|  |  | EXP group (n = 20) | | | CONT group (n = 21) | | |
|  |  | PRE | POST | PRE-to-POST | PRE | POST | PRE-to-POST |
| PT | Extension/flexion | 20 | 20 | **20** | 21 | 21 | **21** |
| ^1^H-MRS | HPC | 18 | 19 | **17** | 19 | 16 | **14** |
|  | SM1 | 20 | 17 | **17** | 21 | 16 | **16** |
|  | dlPFC | 20 | 19 | **19** | 21 | 14 | **14** |
| The values presented in the table are the number (n) of participants with PT data for knee extension-flexion as well as complete, good quality 1H-MRS measurements for each of the voxels after processing with LCModel. The values are presented individually for each time point (PRE and POST) as well as PRE-to-POST which indicates number (n) of participants having complete dataset at PRE as well as POST. Abbreviations: EXP, experimental; CONT, control; PT, peak torque; 1H-MRS, proton magnetic resonance spectroscopy; HPC, hippocampus; SM1, primary sensorimotor cortex; dlPFC, prefrontal cortex | | | | | | | |

| **Supplementary Table** **S3**. Group differences of all test measures | | | | | | |
| --- | --- | --- | --- | --- | --- | --- |
|  |  | EXP | CONT | Total | T-test p- value | Cohen’s d |
| PT knee extension | PRE (20,21) | 117.555 (34.348) | 117.362 (31.701) | 117.456 (32.602) | 0.985 | 0.006 |
|  | POST (20,21) | 123.845 (33.296) | 118.086 (34.975) | 120.895 (33.863) | 0.593 | 0.169 |
| PT knee flexion | PRE (20,21) | 61.575 (16.722) | 62.276 (15.233) | 61.934 (15.779) | 0.889 | 0.044 |
|  | POST (20,21) | 66.37 (14.281) | 59.781 (18.049) | 62.995 (16.458) | 0.204 | 0.405 |
| HPC tNAA/tCr | PRE (18,19) | 1.193 (0.174) | 1.137 (0.100) | 1.164 (0.142) | 0.243 | 0.395 |
|  | POST (19,16) | 1.248 (0.134) | 1.154 (0.175) | 1.205 (0.159) | 0.084 | 0.603 |
| HPC tCho/tCr | PRE (18,19) | 0.323 (0.038) | 0.323 (0.039) | 0.323 (0.038) | 0.964 | 0.000 |
|  | POST (19,16) | 0.331 (0.029) | 0.320 (0.055) | 0.326 (0.042) | 0.468 | 0.250 |
| HPC Glx/tCr | PRE (18,19) | 1.748 (0.386) | 1.554 (0.272) | 1.648 (0.342) | 0.085 | 0.581 |
|  | POST (19,16) | 1.631 (0.221) | 1.677 (0.238) | 1.652 (0.227) | 0.557 | 0.200 |
| HPC mIns/tCr | PRE (18,19) | 1.048 (0.181) | 1.064 (0.169) | 1.056 (0.172) | 0.775 | 0.091 |
|  | POST (19,16) | 1.073 (0.145) | 1.012 (0.235) | 1.045 (0.191) | 0.360 | 0.312 |
| SM1 tNAA/tCr | PRE (20,21) | 1.557 (0.148) | 1.598 (0.143) | 1.578 (0.146) | 0.370 | 0.282 |
|  | POST (17,16) | 1.531 (0.093) | 1.534 (0.151) | 1.533 (0.123) | 0.951 | 0.024 |
| SM1 tCho/tCr | PRE (20,21) | 0.278 (0.039) | 0.266 (0.035) | 0.272 (0.037) | 0.331 | 0.324 |
|  | POST (17,16) | 0.285 (0.036) | 0.273 (0.037) | 0.279 (0.037) | 0.390 | 0.329 |
| SM1 Glx/tCr | PRE (20,21) | 1.052 (0.264) | 1.080 (0.160) | 1.066 (0.214) | 0.677 | 0.128 |
|  | POST (17,16) | 0.962 (0.193) | 0.892 (0.155) | 0.928 (0.177) | 0.258 | 0.400 |
| SM1 mIns/tCr | PRE (20,21) | 0.699 (0.139) | 0.712 (0.098) | 0.706 (0.118) | 0.730 | 0.108 |
|  | POST (17,16) | 0.725 (0.117) | 0.708 (0.134) | 0.717 (0.124) | 0.693 | 0.135 |
| dlPFC tNAA/tCr | PRE (20,21) | 1.363 (0.112) | 1.384 (0.109) | 1.374 (0.110) | 0.547 | 0.190 |
|  | POST (19,14) | 1.341 (0.111) | 1.308 (0.109) | 1.327 (0.110) | 0.411 | 0.300 |
| dlPFC tCho/tCr | PRE (20,21) | 0.315 (0.035) | 0.310 (0.034) | 0.313 (0.038) | 0.689 | 0.145 |
|  | POST (19,14) | 0.317 (0.035) | 0.310 (0.034) | 0.314 (0.034) | 0.536 | 0.203 |
| dlPFC Glx/tCr | PRE (20,21) | 1.236 (0.221) | 1.169 (0.274) | 1.202 (0.249) | 0.393 | 0.269 |
|  | POST (19,14) | 1.078 (0.230) | 1.068 (0.344) | 1.074 (0.279) | 0.915 | 0.034 |
| dlPFC mIns/tCr | PRE (20,21) | 0.837 (0.136) | 0.826 (0.127) | 0.831 (0.130) | 0.803 | 0.084 |
|  | POST (19,14) | 0.805 (0.105) | 0.820 (0.157) | 0.811 (0.128) | 0.754 | 0.112 |
| Values are presented as mean (SD). Values following PRE/POST indicates (EXP(n), CONT(n)), where n is the number of participants having their data for that test measure.  Abbreviations: PT, peak torque HPC, hippocampus; SM1, primary sensorimotor cortex; dlPFC, dorsolateral pre-frontal cortex; tNAA, total N-acetyl aspartate; tCr, total creatine; tCho, total choline; Glx, glutamine-glutamate complex; mIns, myo-inositol | | | | | | |

| **Supplementary Table** **S4**. ANOVA test results for strength parameters based on EXP and CONT group. | | | | |
| --- | --- | --- | --- | --- |
|  |  |  | p-value | Partial Eta Squared |
| PT knee extension | Time | Total | **4.942 (0.032)*** | **0.112** |
|  |  | Experimental | **7.760 (0.008)**** | **0.166** |
|  |  | Control | 0.108 (0.744) | 0.034 |
|  | Group |  | 0.082 (0.776) | 0.002 |
|  | Time*Group |  | 3.112 (0.086) | 0.074 |
| PT knee flexion | Time | Total | 0.564 (0.457) | 0.014 |
|  |  | Experimental | **4.784 (0.035)*** | **0.109** |
|  |  | Control | 1.360 (0.251) | 0.034 |
|  | Group |  | 0.375 (0.544) | 0.010 |
|  | Time*Group |  | **5.665 (0.022)*** | **0.127** |
| Handgrip strength | Time | Total | 2.934 (0.095) | 0.073 |
|  |  | Experimental | 2.698 (0.109) | 0.068 |
|  |  | Control | 0.001 (0.982) | 0.000 |
|  | Group |  | 0.157 (0.694) | 0.004 |
|  | Time*Group |  | 0.027 (0.871) | 0.001 |
| * p < 0.05, ** p < 0.01. Abbreviations: PT, Peak torque | | | | |

| **Supplementary Table** **S5**. Exploratory ANOVA test results for strength parameters based on responders, non-responders and control group. | | | | |
| --- | --- | --- | --- | --- |
|  |  |  | F-value (p-value) | Partial Eta Squared |
| PT knee extension | Time | Total | **16.779 (0.000)***** | **0.306** |
|  |  | Responders | **34.361 (0.000)***** | **0.475** |
|  |  | Non-responders | 0.096 (0.759) | 0.003 |
|  |  | Control | 0.167 (0.685) | 0.004 |
|  | Group |  | 0.213 (0.809) | 0.011 |
|  | Time*Group |  | **13.628 (0.000)***** | 0.418 |
| PT knee flexion | Time | Total | 3.859 (0.057) | 0.092 |
|  |  | Responders | **11.404 (0.002)**** | **0.231** |
|  |  | Non-responders | 0.060 (0.808) | 0.002 |
|  |  | Control | 1.537 (0.223) | 0.039 |
|  | Group |  | 0.341 (0.713) | 0.018 |
|  | Time*Group |  | **6.229 (0.005)**** | **0.247** |
| Handgrip strength | Time | Total | 3.126 (0.086) | 0.080 |
|  |  | Responders | 2.660 (0.112) | 0.069 |
|  |  | Non-responders | 0.003 (0.953) | 0.000 |
|  |  | Control | 1.935 (0.173) | 0.051 |
|  | Group |  | 0.186 (0.831) | 0.010 |
|  | Time*Group |  | 0.780 (0.466) | 0.042 |
| ** p < 0.01, *** p < 0.001. Abbreviations: PT, Peak torque | | | | |

| **Supplementary Table** **S6**. ANOVA test results for all neurometabolite ratios for EXP and CONT group | | | | | |
| --- | --- | --- | --- | --- | --- |
| Brain region | Neurometabolite |  |  | F-value  (p-value) | Partial Eta Squared |
| HPC | tNAA/tCr | Time | Total | 0.865 (0.360) | 0.029 |
|  |  |  | Experimental | 0.973 (0.332) | 0.004 |
|  |  |  | Control | 0.130 (0.721) | 0.032 |
|  |  | Group |  | 3.437 (0.074) | 0.106 |
|  |  | Time*group(2) |  | 0.156 (0.695) | 0.005 |
|  | tCho/tCr | Time | Total | 1.210 (0.280) | 0.040 |
|  |  |  | Experimental | 0.535 (0.471) | 0.018 |
|  |  |  | Control | 0.676 (0.418) | 0.023 |
|  |  | Group |  | 0.007 (0.934) | 0.000 |
|  |  | Time*group(2) |  | 0.014 (0.907) | 0.000 |
|  | Glx/tCr | Time | Total | 0.016 (0.899) | 0.001 |
|  |  |  | Experimental | 2.945 (0.097) | 0.092 |
|  |  |  | Control | 2.995 (0.094) | 0.094 |
|  |  | Group |  | 0.582 (0.452) | 0.020 |
|  |  | Time*group(2) |  | **5.929 (0.021)*** | **0.170** |
|  | mIns/tCr | Time | Total | 0.340 (0.565) | 0.012 |
|  |  |  | Experimental | 0.487 (0.491) | 0.017 |
|  |  |  | Control | 0.024 (0.879) | 0.001 |
|  |  | Group |  | 0.243 (0.626) | 0.008 |
|  |  | Time*group(2) |  | 0.126 (0.725) | 0.004 |
| SM1 | tNAA/tCr | Time | Total | 3.602 (0.067) | 0.104 |
|  |  |  | Experimental | 0.246 (0.623) | 0.008 |
|  |  |  | Control | **4.678 (0.038)*** | **0.131** |
|  |  | Group |  | 1.096 (0.303) | 0.034 |
|  |  | Time*group(2) |  | 1.457 (0.237) | 0.045 |
|  | tCho/tCr | Time | Total | 0.334 (0.568) | 0.011 |
|  |  |  | Experimental | 0.538 (0.469) | 0.017 |
|  |  |  | Control | 0.009 (0.926) | 0.000 |
|  |  | Group |  | 0.529 (0.472) | 0.017 |
|  |  | Time*group(2) |  | 0.197 (0.660) | 0.006 |
|  | Glx/tCr | Time | Total | **8.094 (0.008)**** | **0.207** |
|  |  |  | Experimental | 1.682 (0.204) | 0.051 |
|  |  |  | Control | **7.320 (0.011)*** | **0.191** |
|  |  | Group |  | 0.140 (0.711) | 0.004 |
|  |  | Time*group(2) |  | 1.079 (0.307) | 0.034 |
|  | mIns/tCr | Time | Total | 0.055 (0.817) | 0.002 |
|  |  |  | Experimental | 0.961 (0.335) | 0.030 |
|  |  |  | Control | 0.391 (0.537) | 0.012 |
|  |  | Group |  | 0.000 (0.993) | 0.000 |
|  |  | Time*group(2) |  | 1.279 (0.267) | 0.040 |
| dlPFC | tNAA/tCr | Time | Total | **8.474 (0.007)**** | **0.215** |
|  |  |  | Experimental | 1.216 (0.279) | 0.038 |
|  |  |  | Control | **8.352 (0.007)**** | **0.212** |
|  |  | Group |  | 0.025 (0.876) | 0.001 |
|  |  | Time*group(2) |  | 2.175 (0.150) | 0.066 |
|  | tCho/tCr | Time | Total | 0.031 (0.861) | 0.001 |
|  |  |  | Experimental | 0.013 (0.910) | 0.000 |
|  |  |  | Control | 0.110 (0.743) | 0.004 |
|  |  | Group |  | 0.256 (0.616) | 0.008 |
|  |  | Time*group(2) |  | 0.106 (0.747) | 0.003 |
|  | Glx/tCr | Time | Total | **4.517 (0.042)*** | **0.127** |
|  |  |  | Experimental | 3.399 (0.075) | 0.099 |
|  |  |  | Control | 1.485 (0.232) | 0.046 |
|  |  | Group |  | 0.141 (0.710) | 0.005 |
|  |  | Time*group(2) |  | 0.076 (0.784) | 0.002 |
|  | mIns/tCr | Time | Total | 2.407 (0.131) | 0.072 |
|  |  |  | Experimental | 1.061 (0.311) | 0.033 |
|  |  |  | Control | 1.347 (0.255) | 0.042 |
|  |  | Group |  | 0.227 (0.637) | 0.007 |
|  |  | Time*group(2) |  | 0.044 (0.835) | 0.001 |
| * p < 0.05, ** p < 0.01.  Abbreviations: HPC, Hippocampus; SM1, Primary sensorimotor cortex; dlPFC, dorsolateral pre-frontal cortex; tNAA, total N-acetyl aspartate; tCr, total creatine; tCho, total choline; Glx, glutamine-glutamate complex; mIns, myo-inositol. | | | | | |

| **Supplementary Table** **S7**. Exploratory ANOVA test results for all neurometabolite ratios based on responders, non-responders and control group. | | | | | |
| --- | --- | --- | --- | --- | --- |
| Brain region | Neurometabolite |  |  | F-value  (p-value) | Partial Eta Squared |
| HPC | tNAA/tCr | Time | Total | 1.020 (0.321) | 0.035 |
|  |  |  | Responders | 0.427 (0.519) | 0.015 |
|  |  |  | Non-responders | 0.522 (0.476) | 0.018 |
|  |  |  | Control | 0.126 (0.725) | 0.004 |
|  |  | Group |  | **3.364 (0.049)*** | **0.194** |
|  |  | Time*group(3) |  | 0.080 (0.923) | 0.006 |
|  | tCho/tCr | Time | Total | 2.166 (0.152) | 0.072 |
|  |  |  | Responders | 3.256 (0.082) | 0.104 |
|  |  |  | Non-responders | 0.156 (0.696) | 0.006 |
|  |  |  | Control | 0.719 (0.404) | 0.025 |
|  |  | Group |  | 0.033 (0.968) | 0.002 |
|  |  | Time*group(3) |  | 1.429 (0.256) | 0.093 |
|  | Glx/tCr | Time | Total | 0.195 (0.663) | 0.007 |
|  |  |  | Responders | 0.157 (0.695) | 0.006 |
|  |  |  | Non-responders | 3.331 (0.079) | 0.106 |
|  |  |  | Control | 2.952 (0.097) | 0.095 |
|  |  | Group |  | 0.294 (0.748) | 0.021 |
|  |  | Time*group(3) |  | 3.215 (0.055) | 0.187 |
|  | mIns/tCr | Time | Total | 0.222 (0.641) | 0.008 |
|  |  |  | Responders | 0.025 (0.875) | 0.001 |
|  |  |  | Non-responders | 0.955 (0.337) | 0.033 |
|  |  |  | Control | 0.023 (0.880) | 0.001 |
|  |  | Group |  | **3.669 (0.038)*** | **0.208** |
|  |  | Time*group(3) |  | 0.313 (0.734) | 0.022 |
| SM1 | tNAA/tCr | Time | Total | 1.483 (0.233) | 0.047 |
|  |  |  | Responders | 0.532 (0.471) | 0.017 |
|  |  |  | Non-responders | 1.603 (0.215) | 0.051 |
|  |  |  | Control | **4.811 (0.036)*** | **0.138** |
|  |  | Group |  | 0.987 (0.385) | 0.064 |
|  |  | Time*group(3) |  | 1.690 (0.202) | 0.101 |
|  | tCho/tCr | Time | Total | 0.288 (0.636) | 0.008 |
|  |  |  | Responders | 0.344 (0.562) | 0.011 |
|  |  |  | Non-responders | 2.135 (0.154) | 0.066 |
|  |  |  | Control | 0.009 (0.925) | 0.000 |
|  |  | Group |  | 1.926 (0.163) | 0.114 |
|  |  | Time*group(3) |  | 1.064 (0.358) | 0.066 |
|  | Glx/tCr | Time | Total | **4.658 (0.039)*** | **0.134** |
|  |  |  | Responders | 0.680 (0.416) | 0.022 |
|  |  |  | Non-responders | **6.177 (0.019)*** | **0.171** |
|  |  |  | Control | **8.256 (0.007)**** | **0.216** |
|  |  | Group |  | 0.121 (0.887) | 0.008 |
|  |  | Time*group(3) |  | 3.089 (0.060) | 0.171 |
|  | mIns/tCr | Time | Total | 0.229 (0.635) | 0.008 |
|  |  |  | Responders | 0.029 (0.865) | 0.001 |
|  |  |  | Non-responders | 1.258 (0.271) | 0.040 |
|  |  |  | Control | 0.382 (0.541) | 0.013 |
|  |  | Group |  | 0.328 (0.723) | 0.021 |
|  |  | Time*group(3) |  | 0.799 (0.459) | 0.051 |
| dlPFC | tNAA/tCr | Time | Total | **4.977 (0.033)*** | **0.142** |
|  |  |  | Responders | 0.150 (0.701) | 0.005 |
|  |  |  | Non-responders | 1.149 (0.292) | 0.037 |
|  |  |  | Control | **8.115 (0.008)**** | **0.213** |
|  |  | Group |  | 0.043 (0.958) | 0.003 |
|  |  | Time*group(3) |  | 1.115 (0.341) | 0.069 |
|  | tCho/tCr | Time | Total | 0.002 (0.969) | 0.000 |
|  |  |  | Responders | 0.184 (0.671) | 0.006 |
|  |  |  | Non-responders | 0.035 (0.854) | 0.001 |
|  |  |  | Control | 0.107 (0.746) | 0.004 |
|  |  | Group |  | 0.134 (0.875) | 0.009 |
|  |  | Time*group(3) |  | 0.155 (0.857) | 0.010 |
|  | Glx/tCr | Time | Total | 3.589 (0.068) | 0.107 |
|  |  |  | Responders | 0.044 (0.836) | 0.001 |
|  |  |  | Non-responders | **4.721 (0.038)*** | **0.136** |
|  |  |  | Control | 1.501 (0.230) | 0.048 |
|  |  | Group |  | 0.128 (0.880) | 0.008 |
|  |  | Time*group(3) |  | 0.703 (0.503) | 0.045 |
|  | mIns/tCr | Time | Total | 1.782 (0.192) | 0.056 |
|  |  |  | Responders | 0.116 (0.736) | 0.004 |
|  |  |  | Non-responders | 1.036 (0.317) | 0.033 |
|  |  |  | Control | 1.309 (0.262) | 0.042 |
|  |  | Group |  | 0.234 (0.793) | 0.015 |
|  |  | Time*group(3) |  | 0.082 (0.922) | 0.005 |
| * p < 0.05, ** p < 0.01.  Abbreviations: HPC, Hippocampus; SM1, Primary sensorimotor cortex; dlPFC, dorsolateral pre-frontal cortex; tNAA, total N-acetyl aspartate; tCr, total creatine; tCho, total choline; Glx, glutamine-glutamate complex; mIns, myo-inositol. | | | | | |

| **Supplementary table S8.** The effect of changes (∆) in PT knee extension/flexion after RT on changes (∆) in brain neurometabolite ratios. | | | | | |
| --- | --- | --- | --- | --- | --- |
|  | | EXP | | CONT | |
|  |  | Pearson's r | p-value | Pearson's r | p-value |
| ∆ PT knee extension | ∆ HPC tNAA/tCr | 0.208 | 0.422 | -0.168 | 0.566 |
|  | ∆ HPC Glx/tCr | 0.047 | 0.858 | -0.478 | 0.084 |
|  | ∆ HPC tCho/tCr | 0.336 | 0.187 | -0.083 | 0.779 |
|  | ∆ HPC mIns/tCr | 0.085 | 0.745 | -0.325 | 0.257 |
|  | ∆ SM1 tNAA/tCr | **0.540** | **0.025*** | 0.308 | 0.246 |
|  | ∆ SM1 Glx/tCr o.e | **0.716** | **0.002**** | -0.219 | 0.415 |
|  | ∆ SM1 tCho/tCr | -0.025 | 0.923 | -0.015 | 0.956 |
|  | ∆ SM1 mIns/tCr | 0.016 | 0.952 | -0.040 | 0.884 |
|  | ∆ dlPFC tNAA/tCr | 0.026 | 0.916 | 0.015 | 0.961 |
|  | ∆ dlPFC Glx/tCr o.e | **0.637** | **0.006**** | -0.255 | 0.379 |
|  | ∆ dlPFC tCho/tCr | -0.097 | 0.693 | -0.581 | 0.029 |
|  | ∆ dlPFC mIns/tCr | 0.291 | 0.227 | -0.144 | 0.623 |
| ∆ PT knee flexion | ∆ HPC tNAA/tCr | 0.058 | 0.825 | -0.008 | 0.977 |
|  | ∆ HPC Glx/tCr | 0.208 | 0.423 | -0.111 | 0.706 |
|  | ∆ HPC tCho/tCr | 0.055 | 0.833 | **0.558** | **0.038*** |
|  | ∆ HPC mIns/tCr | -0.079 | 0.765 | -0.142 | 0.629 |
|  | ∆ SM1 tNAA/tCr | **0.544** | **0.024*** | -0.016 | 0.952 |
|  | ∆ SM1 Glx/tCr o.e | **0.593** | **0.016*** | -0.283 | 0.289 |
|  | ∆ SM1 tCho/tCr | 0.112 | 0.668 | -0.152 | 0.574 |
|  | ∆ SM1 mIns/tCr | 0.317 | 0.215 | -0.062 | 0.819 |
|  | ∆ dlPFC tNAA/tCr | 0.329 | 0.097 | 0.133 | 0.649 |
|  | ∆ dlPFC Glx/tCr o.e | **0.509** | **0.037*** | -0.046 | 0.876 |
|  | ∆ dlPFC tCho/tCr | 0.162 | 0.508 | -0.311 | 0.280 |
|  | ∆ dlPFC mIns/tCr | **0.635** | **0.003**** | -0.245 | 0.364 |
| **p < 0.01; *p < 0.05 Single linear regression analysis for ∆ PT of knee movement. Abbreviations: PT, peak torque; dlPFC, dorsolateral prefrontal cortex; HPC, hippocampal cortex; SM1, sensorimotor cortex; Glx, glutamine-glutamate complex; o.e., influential outlier excluded; mIns, myoinositol; tCho, total choline; tCr, total creatine, tNAA, total N-acetyl aspartate. | | | | | |

|  | | | | | |
| --- | --- | --- | --- | --- | --- |
| **Supplementary Table** **S9.** The effect of changes (∆) in regional tNAA/tCr on other brain neurometabolite ratios in EXP and CONT group | | | | | |
|  | | EXP | | CONT | |
|  |  | Pearson’s r | p-value | Pearson’s r | p-value |
| ∆ HPC tNAA/tCr | ∆ HPC Glx/tCr | 0.278 | 0.280 | 0.034 | 0.908 |
|  | ∆ HPC tCho/tCr | 0.448 | 0.072 | -0.043 | 0.885 |
|  | ∆ HPC mIns/tCr | **0.694** | **0.002**** | 0.051 | 0.865 |
|  | ∆ SM1 tNAA/tCr | 0.051 | 0.850 | 0.191 | 0.512 |
|  | ∆ SM1 Glx/tCr o.e | 0.012 | 0.967 | -0.152 | 0.604 |
|  | ∆ SM1 tCho/tCr | 0.311 | 0.241 | **-0.616** | **0.019*** |
|  | ∆ SM1 mIns/tCr | -0.326 | 0.218 | -0.303 | 0.292 |
|  | ∆ dlPFC tNAA/tCr | 0.120 | 0.646 | 0.403 | 0.194 |
|  | ∆ dlPFC Glx/tCr o.e | -0.080 | 0.777 | -0.398 | 0.201 |
|  | ∆ dlPFC tCho/tCr | 0.092 | 0.726 | 0.419 | 0.175 |
|  | ∆ dlPFC mIns/tCr | -0.023 | 0.931 | -0.078 | 0.810 |
| ∆ SM1 tNAA/tCr | ∆ HPC Glx/tCr | -0.094 | 0.730 | -0.158 | 0.590 |
|  | ∆ HPC tCho/tCr | 0.079 | 0.771 | -0.065 | 0.826 |
|  | ∆ HPC mIns/tCr | 0.032 | 0.905 | 0.061 | 0.836 |
|  | ∆ SM1 Glx/tCr o.e | **0.673** | **0.004**** | -0.478 | 0.061 |
|  | ∆ SM1 tCho/tCr | 0.385 | 0.127 | 0.450 | 0.080 |
|  | ∆ SM1 mIns/tCr | 0.186 | 0.475 | **0.554** | **0.026*** |
|  | ∆ dlPFC tNAA/tCr | 0.187 | 0.474 | 0.372 | 0.190 |
|  | ∆ dlPFC Glx/tCr o.e | **0.574** | **0.025*** | -0.211 | 0.470 |
|  | ∆ dlPFC tCho/tCr | 0.016 | 0.952 | 0.076 | 0.798 |
|  | ∆ dlPFC mIns/tCr | 0.112 | 0.667 | 0.308 | 0.285 |
| ∆ dlPFC tNAA/tCr | ∆ HPC Glx/tCr | 0.026 | 0.922 | 0.359 | 0.252 |
|  | ∆ HPC tCho/tCr | -0.162 | 0.534 | 0.521 | 0.082 |
|  | ∆ HPC mIns/tCr | -0.214 | 0.409 | **0.618** | **0.032*** |
|  | ∆ SM1 Glx/tCr o.e | 0.297 | 0.264 | -0.161 | 0.581 |
|  | ∆ SM1 tCho/tCr | -0.326 | 0.202 | 0.155 | 0.596 |
|  | ∆ SM1 mIns/tCr | 0.096 | 0.713 | -0.044 | 0.881 |
|  | ∆ dlPFC Glx/tCr o.e | -0.025 | 0.924 | -0.524 | 0.054 |
|  | ∆ dlPFC tCho/tCr | 0.303 | 0.207 | 0.223 | 0.443 |
|  | ∆ dlPFC mIns/tCr | **0.562** | **0.012*** | 0.085 | 0.772 |
| **p < 0.01; *p < 0.05. Single linear regression analysis for regional ∆ tNAA/tCr. Abbreviations: dlPFC, dorsolateral prefrontal cortex; HPC, hippocampal cortex; SM1, sensorimotor cortex; Glx, glutamine-glutamate complex; o.e, influential outlier excluded; mIns, myoinositol; tCho, total choline; tCr, total creatine, tNAA, total N-acetyl aspartate. | | | | | |

| **Supplementary table S10.** The effect of changes (∆) in PT knee extension/flexion after RT on changes (∆) in brain neurometabolite ratios based on responders and non-responders | | | | | |
| --- | --- | --- | --- | --- | --- |
|  | | Responders | | Non-responders | |
|  |  | Spearman's ρ | p-value | Spearman's ρ | p-value |
| ∆ PT knee extension | ∆ HPC tNAA/tCr | **0.829** | **0.042*** | 0.409 | 0.212 |
|  | ∆ HPC Glx/tCr | 0.314 | 0.544 | -0.245 | 0.467 |
|  | ∆ HPC tCho/tCr | 0.086 | 0.872 | 0.027 | 0.937 |
|  | ∆ HPC mIns/tCr | **0.943** | **0.005**** | 0.273 | 0.417 |
|  | ∆ SM1 tNAA/tCr | **0.736** | **0.036*** | 0.152 | 0.676 |
|  | ∆ SM1 Glx/tCr o.e | 0.750 | 0.052 | 0.133 | 0.732 |
|  | ∆ SM1 tCho/tCr | 0.571 | 0.180 | 0.515 | 0.128 |
|  | ∆ SM1 mIns/tCr | 0.500 | 0.253 | -0.333 | 0.347 |
|  | ∆ dlPFC tNAA/tCr | 0.429 | 0.337 | -0.217 | 0.499 |
|  | ∆ dlPFC Glx/tCr o.e | **0.943** | **0.005**** | 0.191 | 0.574 |
|  | ∆ dlPFC tCho/tCr | -0.107 | 0.819 | -0.189 | 0.557 |
|  | ∆ dlPFC mIns/tCr | **0.821** | **0.023*** | -0.105 | 0.746 |
| ∆ PT knee flexion | ∆ HPC tNAA/tCr | 0.543 | 0.266 | 0.236 | 0.484 |
|  | ∆ HPC Glx/tCr | -0.029 | 0.957 | **0.736** | **0.010*** |
|  | ∆ HPC tCho/tCr | -0.143 | 0.787 | 0.264 | 0.433 |
|  | ∆ HPC mIns/tCr | 0.657 | 0.156 | -0.082 | 0.811 |
|  | ∆ SM1 tNAA/tCr | **0.964** | **0.000***** | 0.176 | 0.627 |
|  | ∆ SM1 Glx/tCr o.e | **0.857** | **0.014*** | 0.117 | 0.765 |
|  | ∆ SM1 tCho/tCr | **0.857** | **0.014*** | -0.164 | 0.651 |
|  | ∆ SM1 mIns/tCr | 0.643 | 0.119 | 0.370 | 0.293 |
|  | ∆ dlPFC tNAA/tCr | 0.214 | 0.645 | 0.315 | 0.319 |
|  | ∆ dlPFC Glx/tCr o.e | 0.543 | 0.266 | -0.036 | 0.915 |
|  | ∆ dlPFC tCho/tCr | 0.250 | 0.589 | 0.091 | 0.779 |
|  | ∆ dlPFC mIns/tCr | **0.893** | **0.007**** | 0.476 | 0.118 |
| **p < 0.01; *p < 0.05 Single linear regression analysis for ∆ PT of knee movement. Abbreviations: PT, peak torque; dlPFC, dorsolateral prefrontal cortex; HPC, hippocampal cortex; SM1, sensorimotor cortex; o.e., influential outlier excluded; Glx, glutamine-glutamate complex mIns, myoinositol; tCho, total choline; tCr, total creatine, tNAA, total N-acetyl aspartate. | | | | | |

**Supplementary Fig 1.** Study design and intervention protocol of resistance training for experimental group participants


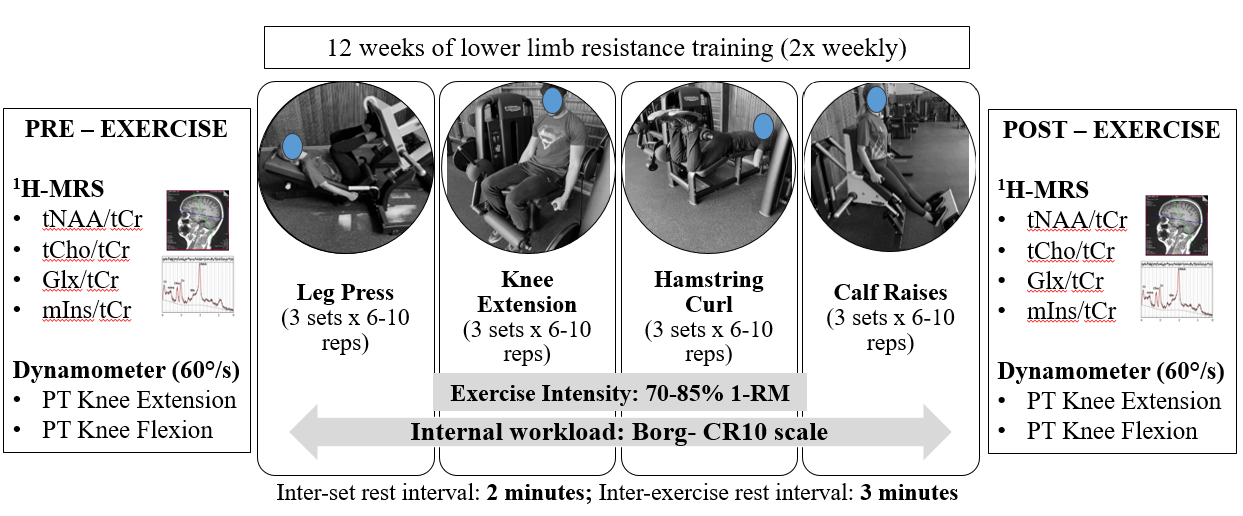


Abbreviations: ^1^H-MRS, magnetic resonance spectroscopy; tNAA, total N-acetyl aspartate; tCr, total creatine; tCho, total choline; Glx, glutamine-glutamate complex; mIns, myo-inositol.

**Supplementary Fig 2.** Linear relationship between changes in strength levels (PT knee extension and flexion) and neurometabolite ratios in HPC, SM1 and dlPFC (tNAA/tCr, Glx/tCr, tCho/tCr and mIns/tCr)


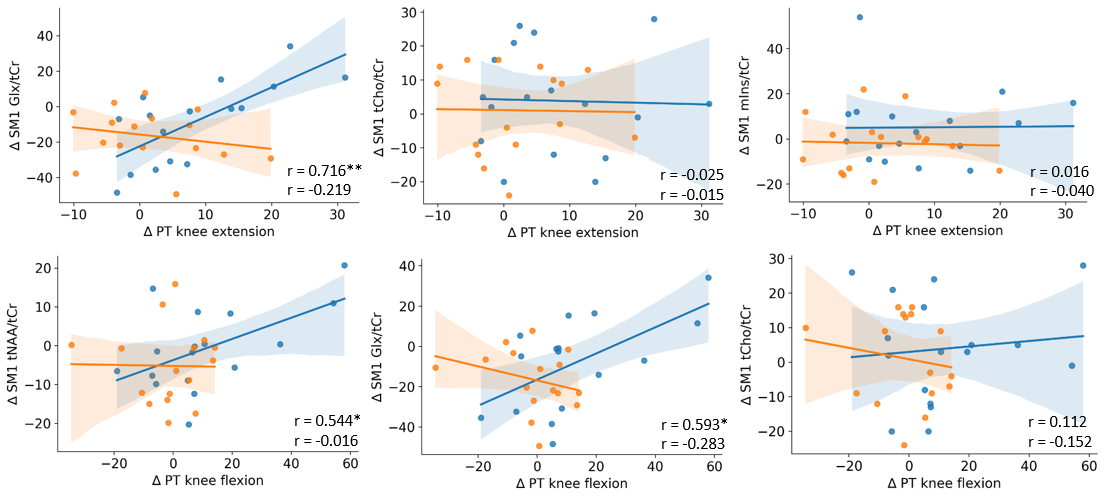

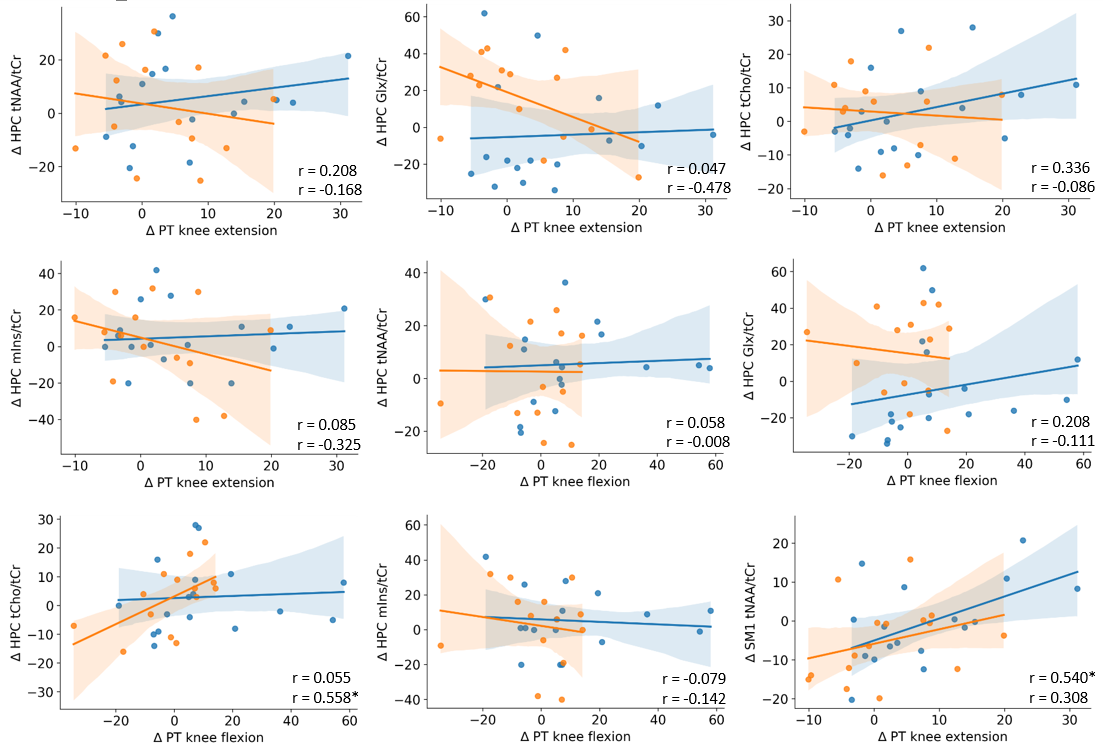


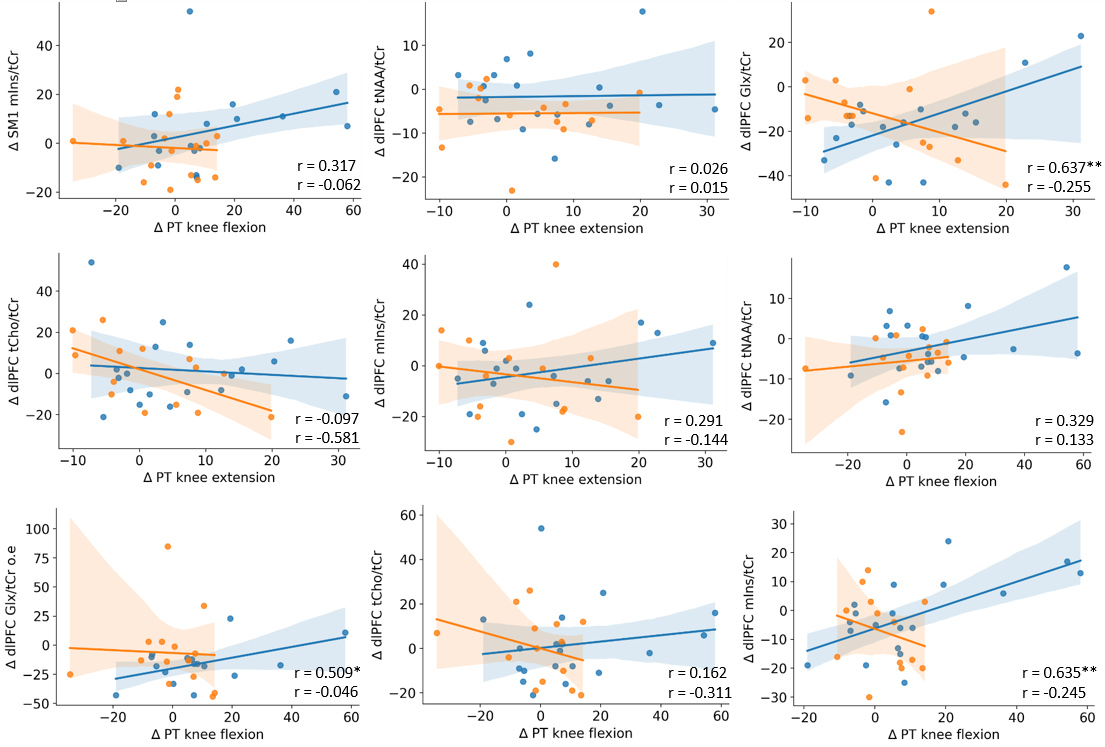

Supplement: Supplementary file 1 — Supplementary file1 (DOCX 833 KB) [file 11357_2023_732_MOESM1_ESM.docx]
